# Food industry political practices in Chile: “the economy has always been the main concern”

**DOI:** 10.1186/s12992-020-00638-4

**Published:** 2020-10-27

**Authors:** Mélissa Mialon, Camila Corvalan, Gustavo Cediel, Fernanda Baeza Scagliusi, Marcela Reyes

**Affiliations:** 1 School of Public Health, University of São Paulo, Av. Dr. Arnaldo, 715 - Cerqueira César, São Paulo, SP 01246-904 Brazil; 2 Institute of Nutrition and Food Technology (INTA), University of Chile, El Líbano, 5524 Macul, Región Metropolitana Chile; 3 School of Nutrition and Dietetics, University of Antioquia, Cra. 74a, #93-30 Medellín, Colombia

**Keywords:** Commercial determinants of health, Corporate political activity, Food industry, Non-communicable diseases

## Abstract

**Background:**

In the business literature, the term “corporate political activity” (CPA) refers to the political strategies undertaken by corporations to protect or expend their markets, by influencing, directly or indirectly, the policy process. There is evidence that food industry actors use such political practices, which poses a significant threat to public health. Our study objective was to identify the political practices of the food industry in Chile.

**Results:**

In Chile, food industry actors supported community initiatives, particularly those targeted at children and those focused on environmental sustainability. Food industry actors also funded research through prizes, scholarships, and by supporting scientific events. Food industry actors lobbied against the development and implementation of a front-of-pack nutrition labelling policy, including with support from the Ministries of Economy, Agriculture and Foreign Affairs. Food industry actors, for example, claimed that there would be unintended negative consequences for society and the economy, and that the policy would breach trade agreements. The same arguments were used against a proposed tax increase on sugar-sweetened beverages. Food industry actors stressed their crucial role in the Chilean economy and claimed to be part of the solution in the prevention and control of obesity, with a particular focus on their efforts to reformulate food products, and their support of physical activity initiatives. Interviewees noted that the political influence of the food industry is often facilitated by the neo-liberal and market-driven economy of Chile. Nevertheless, this system was questioned through social protests that started in the country during data collection.

**Conclusions:**

In Chile, food industry actors used numerous action- and argument-based CPA practices which may influence public health policy, research, and practice. Despite strong influence from the food industry, Chile adopted a front-of-pack nutrition labelling policy. While the country has some measures in place to manage the interactions between government officials or public health professionals, and the industry, there is still a need to develop robust mechanisms to address undue influence from corporations.

**Supplementary Information:**

The online version contains supplementary material available at 10.1186/s12992-020-00638-4.

## Background

In Chile, like many other countries around the globe, unhealthy diets, overweight, and obesity are the leading risks factors of disability and death [1]. Chile was one of the first countries to adopt, in 2012, a comprehensive policy to address these issues, known as Law 20.606, that focused on the ‘nutritional composition of foods and their advertising’, that we will here refer to as the ‘front-of-pack labelling’ (FOPL) policy [2–4]. Peru and Uruguay have since then introduced similar policies [5], and other countries across the globe are currently considering adopting a new FOPL policy. In Chile, Law 20.606 was discussed in the Congress between 2007 and 2012; the Ministry of Health developed the Decree between 2012 and 2015; and the policy was implemented in a staggered manner between 2016 and 2019 [6]. During that time, different political coalitions were leading the Chilean government. Law 20.606 mandates the use of warning labels on the front of food products that have an excess of calories and sugars, sodium and fats [2]. It also restricts the marketing of such products to children [2]. In the face of continuous rising levels of obesity, the Council on Healthy Development was also launched in October 2019 to propose solutions and recommendations to address this epidemic [7].

It has been reported that the food industry lobbied against the development of this Law in Congress, claiming that it was a breach of personal freedom, and proposed an alternative FOPL system, and, as a consequence, the Law was discussed in Congress for over a decade before it was fully implemented [8]. Two transnationals, PepsiCo and Kellogg’s, also challenged the Law in the court [9]. Similar arguments were documented in other parts of the globe during the development of new FOPL systems [3, 10–12]. In parallel, in 2014, an increase in the taxation of sugar-sweetened beverages was proposed in Chile but rapidly watered down, due to industry opposition [13]. In this case, the industry claimed that there was a lack of evidence to support the policy and that the tax would be a barrier to the economic development of the country [13].

In the business literature, ‘corporate political activity’ (CPA) refers to the activities that corporations undertake to protect or expend their markets, by influencing, directly or indirectly, the policy process [14]. Beyond the direct involvement and influence on policy of businesses, the (CPA) could be exerted through: interactions with third parties (coalition management); information shaping (information management); legal strategies; and discursive strategies [15]. These five strategies are presented in Additional file 1. In public health, these practices were first studied for the tobacco industry, after litigation against large American-based tobacco companies in the late 1990s and the compulsory release of millions of pages of internal documents [16, 17]. There is ample evidence that the political influence of the tobacco industry results in negative impacts on public policy, and, ultimately, on population health [18]. This has led the World Health Organization to include an article stating that countries should protect public health policies from the influence of the tobacco industry in its Framework Convention on Tobacco Control [19]. The CPA of the food industry, aimed at protecting or increasing the sales of foods, including unhealthy products, also poses a significant threat to public health [20, 21]. Such threats were documented in several parts of the globe [9, 22–25]. However, beyond the anecdotal evidence cited above, little is known about the political influence of the food industry in Chile. With the present study, we aimed to identify the CPA strategies of the food industry in Chile.

## Methods

Data collection included a document analysis as well as interviews with informants working in public health nutrition in Chile and was carried out in November and December 2019. All data were collected in Spanish by the first author, who has working proficiency in that language and experience conducting qualitative research. All researchers worked in public health at the time of data collection and took a critical approach to the influence of the food industry on public health policy.

This study was part of a large project on the CPA of the food industry in Latin America and was approved by the ethics committee of the School of Public Health at the University of Sao Paulo, Brazil (project number 07944118.7.0000.5421). The fieldwork in Chile was approved locally by the ethics committee at INTA. Two authors of the present manuscript work at INTA, but have no interaction with the food industry. No one at INTA, except from these two researchers, influenced the design, conduct and reporting of the present study.

### Data available in the public domain

For our document analysis, we followed the protocol of INFORMAS (International Network for Food and Obesity/non-communicable diseases Research, Monitoring and Action Support) for collecting CPA information [26]. These methods are described in details elsewhere and consist of an analysis of publicly available data [26]. Given our time constraints and based on the results of a pilot study on the CPA of the food industry in Latin America [9], in addition to an initial analysis of the websites of Nestlé and PF Alimentos in Chile (random selection of industry actors amongst our sample), we decided to restrict our searches to data published during the period 1st January - 31st December 2019. Some information nevertheless referred to events or activities that took place before 1st January (e.g.; a Twitter post from January 2019 that referred, retrospectively, to an event from December 2018).

As recommended by INFORMAS, we collected information on the most prominent food industry actors in Chile. Given that we had no access to the market shares of these actors, inclusion was decided based on discussion with local experts. Our final sample included the twelve members of the International Food and Beverage Alliance (IFBA): Coca-Cola, Danone, Ferrero/AgriChile, General Mills, Grupo Bimbo/Ideal, Kellogg’s, Mars, McDonald’s, Mondelez, Nestlé, PepsiCo, Unilever). This coalition represents the largest food manufacturers, globally, in terms of market shares. We also included three Chilean food manufacturers (Carozzi Alimentos; PF Alimentos; Soprole); one association in food science and technology (the Asociación Chilena de Ciencia y Tecnología en Alimentos (Sochital, the Chilean Association of Food Science and Technology); one research institute funded by the food industry (The International Life Science Institute - ILSI Sur-Andino); and three trade associations (Chile Alimentos; the Asociación Gremial De Alimentos Y Bebidas De Chile A.G. - AB Chile (Guild Association of Food and Drinks of Chile); the Supermercados de Chile A. G (‘Supermarkets of Chile’).

For each of these actors, when available, we consulted their national website(s) and Twitter account(s). As recommended by INFORMAS, in addition to the industry’s webpages, we collected information from universities with a curriculum in nutrition, from nutrition professional associations, and the Chilean Congress, Ministries and other sources from the government. These sources and websites are listed in Additional file 2. All data collected from the public domain is available as an Additional file 3 (data is in Spanish). Danone, General Mills, Grupo Bimbo/Ideal, Mars, and Mondelez had no national websites or Twitter accounts. The website of Sochital was not working during the period of data collection.

### Interviews

In addition to the analysis of publicly available information, we conducted interviews. Our interviewees described CPA examples for the food industry, but with no restriction on the timeframe for these examples to have happened and no focus on specific industry actors (compared to our document analysis where we had a sample of specific actors). Our sampling was purposive, and we identified, through our professional networks, individuals who had interacted with the food industry and observed the CPA of the food industry, as discussed in the media or other online material. Our interviewees then identified other potential participants (snowball sampling). The interviewer had no existing relationship with the interviewees and the interviewees had no knowledge of the interviewer. We interviewed current and former members of the government involved in the development or implementation of nutrition policies (*n* = 6) and academics working in public health nutrition (*n* = 3).

Participants were contacted by emails and received a copy of the ethics consent form, which was signed at the beginning of the interview. The interviews were conducted face-to-face (and on Skype for one follow-up interview) at a place chosen by the interviewees. They lasted 1 hour on average and were digitally recorded with the consent of participants. The transcription was done by a contracted professional, under the strict condition of confidentiality. Interviewees could revise the copy of their transcript before the submission of the present manuscript. The interview guide is available in Additional file 4. The interviewer determined that saturation was reached when no new CPA practice was discussed and when, within the different CPA practices, participants started to share examples that were already discussed by other interviewees.

### Data analysis and reporting

For both the document analysis and interviews, we used the recommendations of INFORMAS for data analysis and undertook a deductive approach, led by the first author, and used an existing framework for classifying the CPA of the food industry (Additional file 1). Microsoft Excel and Word were used to manage all data. The findings were constantly discussed over the life of the project between the first, second and last authors. The third author reviewed 10% of the coding and agreement was reached through discussion between the first and third authors (not quantified).

We found new CPA practices and modified the framework from Additional file 1 during data analysis in an iterative process, and using the existing literature on the CPA of the tobacco industry [15]. New codes included:

- Coalition management - we added a category “internal” when industry actors built alliances within other actors in the industry;
- Direct involvement and influence in policy, indirect access - we added the code “co-opt government officials” when government officials were representing the food industry;
- Discursive strategies – we added the code “unanticipated costs to the economy and society” when industry actors discussed the loss of sales, breaches of trade agreements and other costs beyond those on the industry itself;
- Discursive strategies – we also added the code “intended public health benefits” when the industry claimed that a policy would not have the intended benefits on health or moderate benefices only.

For reporting our research, we followed the COnsolidated criteria for REporting Qualitative research (COREQ) (see Additional file 5). We use, in the results section, a code starting with the letter A to refer to data from the public domain, as presented in Additional file 3. For the interviews, we have removed sensitive information that could identify our participants, and only use generic identifiers such as “her” or “she” when reporting our findings. For interviewees from the government, and to protect their anonymity, we refer to ‘members of the government’ with no distinction between those who left the government and those who still work in it. We used illustrative examples in the results section and the CPA framework for guiding our writing of that section.

### A note on the context for data collection in this study

When starting her fieldwork, in September 2019, local experts warned the lead researcher of the study (and first author) that industry influence on policy was not necessarily questioned in Chile. They noted that there was little civil society engagement in public health nutrition. Also, they highlighted that most researchers and health professionals do not question collaborations with the food industry in Chile. On 18 October 2019, a massive protest started in the capital city, Santiago, and rapidly spread to the rest of the country, after an increase in the price of public transport. The few days that followed were chaotic, with a state of emergency, curfews, and destruction, particularly in Santiago, where this research took place. These protests had a direct impact, not only during the fieldwork for this study but also on the results presented in this manuscript.

First, some interviews were conducted in neighbourhoods where the protests were happening, in places chosen by our interviewees, which represented a risk for the safety of both participants and the interviewer. In addition, three individuals who accepted to participate in this study could not be interviewed because it was either impossible to schedule an interview due to issues with transportation, because some public transports like buses and metros were destroyed; or because some places in the city were unsafe to meet; or because interviewees were extremely busy participating in the social movement or the policy changes in Congress, in direct response to the protests. These conditions mean that, for this study, we were not able to interview journalists or public health advocates (those outside academia and government).

Secondly, some interviewees expanded the discussion on the CPA of the food industry, and shared their perspectives on the influence of corporations more broadly on public health policy, research and practice, as this was now questioned in Chile. Their opinions were beyond the initial scope of the study but became part of our research. These perspectives are therefore presented in our results.

## Results

We identified 130 examples of CPA practices during our analysis of publicly available information: 92 examples of instrumental strategies and 77 examples of discursive strategies (the categories are non-mutually exclusive as one example could belong to both categories). Our interviewees also discussed some of the examples identified in the analysis of publicly available information and identified additional CPA practice, as discussed below. Several participants in our interviews, particularly those who work/worked in the government, described the CPA of the food industry during the development of Law 20.606 (‘the FOPL policy’), probably because this was a prominent topic for many years in nutrition in the country.

### Coalition management

We identified examples of coalition management, where the food industry tried to build alliances with third parties, or, on the contrary, tried to discredit public health advocates and other critiques. We collected several examples of initiatives undertaken by different actors in the food industry in Chile in 2019 (see Table 1, non-exhaustive; for more details, see Additional file 3). Many of these initiatives were targeted at children and schools, and centred on the issue of environmental sustainability. It is important to note that, in Chile, sustainability was often used by companies to promote their products, including those that may be considered unhealthy. For example, producers of sugar-sweetened beverages and packaged foods have organised for years visits of their factories for students, including their production lines, as well as family runs, with the distribution of water in plastic bottles. In another example, Nestlé inaugurated a new “Eco-Zone” in a museum, where the logo of the company was prominently displayed [A91 Nestlé].

**Table 1:** Coalition management strategy - community initiatives of the food industry in Chile in 2019 (non-exhaustive)

Food industry actor	Initiative	Nature of the event and targeted population	Partner, when applicable	Interviewee or code from Additional file 3
Carozzi	Carozzi Kids Challenge	Physical activity, children		A11
	Visit of a factory for students in secondary schools and universities *“that allow them to know (…) the preparation of products such as our Cereal Bar. They also face a scanner that indicates which portions should be consumed during the day as well as participating in sports letter soups.”*	Nutrition, students in secondary schools and universities	The Institute of Nutrition and Food Technology (INTA) prepared a talk on healthy eating and active lifestyle	A18
Chile Alimentos	Chile Crece Sano (Chile Grows Healthy)	Nutrition, general public		A24
Coca-Cola	Visit of a factory (including the production line) for students	Sustainability, students		A43, A47
	Foundation “Fútbol Más” in schools	Physical activity, children		A48
	Family run, with the distribution of plastic bottles and education on how to recycle	Physical activity and sustainability, families		A49
	A discussion about women in society	Gender, women	Presence of Members of Parliament and officials of the Ministry of Sciences	A46
	Two days of trekking and education on sustainability for university students	Students in universities	The charity Nature Conservancy	A56
	A campaign for protecting the environment	Sustainability, general public	Ministry of Environment	Interview, member of the government
Fundación Arcos (sweets)	Schools on the move	Physical activity, students		Interview, member of the government
Mc Donald’s	Campaign Big Mac heart	Support for children in hospitals	Coanil Foundation and the Ronald McDonald Foundation for Children	A64
Nestlé	Local Development Fund, Henri Nestlé Award 2019	Sustainability and nutrition, general public	Targeted at “*community organisations (…) from the municipalities where Nestlé Chile operates through its factories*”	A89
	Programme Nestlé Niños Saludables	Nutrition, children	All the contents are gathered in a Didactic Guide for the Teacher, sponsored by the Ministry of Education. “*The Nestlé Healthy Children program is sponsored by three important institutions, which jointly reviewed and validated the contents of this Guide: INTA, Fundación 5 al Día and the Chilean Society of Nutrition (SOCHINUT).*”	A112
PepsiCo	Donation of sports kits for students	Physical activity, students	Ministry of Sport and famous athletes	A116
	Training sessions for teachers and parents, workshops for children and the delivery of sports equipment	Nutrition and physical activity, children, teachers & parents		A118
	Live Healthy Workshops	Nutrition and physical activity, general public		A120
	Programme “Let’s play with our children”	Leisure, children	Universidad Católica de Chile	A121
	Not stated	N/A	UChile, FAO, the government of Chile, the programme “Elige Vivir Sano”	A123

### Information management

During data collection, we found evidence of the influence of the food industry on the science and information published and disseminated on the topic of nutrition.

The sweeteners industry was, for example, in the context of food reformulation after the implementation of the FOPL policy, interacting with the academic community in Chile through scientific presentations around the country in 2019 where it promoted its products [A2 International Sweeteners Association].

*“This gave rise to a renewed interest in developing research on sweeteners other than sugar, and within this framework, the First Conference on Sweeteners was held in Santiago, a talk for professionals convened by the Nutrition and Dietetics Program at the Universidad Santo Tomás.” [A52 Coca-Cola]*

*"[In the] School of Nutritionists [ … ] they defend sweeteners, they have classes about sweeteners everywhere, they travel around the country talking about sweeteners." [member of government]*

Moreover, since 2005 Nestlé funds a prize for researchers in nutrition, in partnership with the Sociedad Chilena de Nutrición (Chilean Nutrition Society, SOCHINUT).

*"In the almost 15 years of history of the SOCHINUT - Henri Nestlé recognition, nearly 30 researchers have been recognised, with projects that have contributed to having more and better information on the nutritional properties of foods and their potential in the prevention of some diseases. On this occasion, the projects were evaluated by a committee of judges made up of experts, the SOCHINUT board and a committee of Nestlé experts." [A82, A108 Nestlé]*

In addition, since 1998, Nestlé sponsored a scholarship for the students of INTA, which current objective is to “*contribute to the academic and institutional development of INTA, through the financial support of maintenance for young researchers who show interest and potential to start an academic career*” [A87 Nestlé].

Several interviewees shared examples where companies in the food industry paid for studies in public health nutrition and, on some occasions, suppressed the results, or withdraw their funding to the institutions that had published unfavourable results for the industry.

*"When they see that there is a researcher who is entering an area that could be potentially threatening ... they absorb him, incorporate him into their networks, through hiring or offer you things." [academic]*

*"Some researchers (...) worked on [a product], and it turned out that [the product] they were studying did not work, and the researchers published the results anyway. That means that the company (...) took out the money that they gave [to the university]." [academic]*

*"A colleague had done a study, and the results were not pleasing the industry, and they were expressly asked to change the results. (...) They asked her to falsify the data." [member of the government]*

In our interviews and document analysis, we identified a list of events organised or sponsored by the food industry in 2019 (Table 2, non-exhaustive) (amplification).

**Table 2:** Information management - scientific events with the participation of the food industry in 2019 in Chile (non-exhaustive)

Event	Food industry actor	Nature of the participation for the food industry	Code from Additional file 3
X Chilean Congress of Clinical Nutrition, Obesity and Metabolism, III Congress of Paediatric Clinical Nutrition	Nestlé	Sponsor	A86
ILSI SurAndino IV Conference on Nutrition and Dietetics: “Nutrition and evidence-based nutrition: implications in cancer”	ILSI	Organiser	A69
Research Conference on Nutrition and Food	ILSI	Organiser	A72
Series of lectures for the students in Nutrition and Dietetics at the Universidad Santo Tomás and Universidad SEK	ILSI	Organiser	A76–8
Updates in Clinical Nutrition at the Clínica Alemana Temuco	Nestlé	Sponsor	A83
IV Course of Nutritionists up to date at Clinica Las Condes, organized by the Clinica Las Condes, and the Department of Nutrition, Faculty of Medicine, University of Chile	Nestlé	Sponsor	A84
8th Celiac Disease Symposium, organised by INTA and COACEL (Celiac Support Corporation)	PF Alimentos	Participant	A124
3rd version “Dairy, nutrition and health course: What does the scientific evidence tell us?” at the Faculty of Medicine, University of Chile	Consorcio Lechero (which includes Nestlé and Soprole)	Organiser	A127

Several scientific events presented in Table 2 were organised by the South Andean branch of ILSI (the International Life Science Institute). This research institute claimed to be independent but has been criticised in recent years for representing the interests of its funders, large transnationals in the agribusiness and food industry [27, 28]. In Chile, the companies that are members of ILSI are Coca-Cola, Danisco-Dupont, DSM, Kraft, Monsanto, Nestlé, Tres Montes Luchetti, and Unilever [A79 ILSI].

In our study, we found evidence that food industry actors in Chile had relationships with several universities (Tables 1 and 2). Carozzi and Nestlé, for example, got support from the INTA to develop nutrition education material (Table 1). Moreover, in October 2019, INTA signed a partnership with a consortium of milk manufacturers, representing companies such as Nestlé and Soprole, to promote the consumption of dairy in the population.

*“The alliance seeks to enhance the activities and technical capacities of each institution, to promote the benefits of milk among consumers, health professionals and authorities, through research and extension projects, among others. ( … .) This public-private alliance reflects the interest in generating instances to publicise the benefits of milk to consumers, health professionals, and authorities, through cooperation between the two entities. "Given the worrying increase in diseases such as obesity, diabetes or cardiovascular diseases, dairy products appear as an easily accessible food for most people, fast and easy to consume in their different presentations. Natural and flavoured milk, yogurt, cheese, are options that must be part of a balanced and healthy diet", said [the] director of the Technical Assistance office at INTA.” [A129]*

These relations between the industry and universities had a direct impact on our study, with, two individuals telling us that they refused to participate in our interviews because our research was conducted in collaboration with INTA and they felt our study on the CPA of the food industry was not credible because of the work INTA does with some food companies.

### Direct involvement and influence in policy and legal strategies

Our interviewees discussed the direct involvement and influence of the food industry on the development of the FOPL policy. They mentioned the lobbying that happened in Congress and in *La Moneda*, where the President of the Republic sits. Our participants mentioned that some former members of the government were working for the food industry during the development of the FOPL policy (i.e., they used what is called ‘the revolving door’), and other members of the government who had conflicts of interest, actively participated in lobbying against the proposed legislation.

*“The spokesperson for AB Chile [was] Minister of [Finance], during the Piñera period, he had been a parliamentarian, ( … ) a Member of Parliament for twelve years. ( … ) [He] lobbied the other authorities.” [member of the government]*

*“More than just campaigning, Chile Alimentos was going to express its concern directly with [high government officials].” [member of the government]*

Interviewees noted that the Ministries of Economy, Agriculture and Foreign Affairs also lobbied against the FOPL policy, often repeating the same arguments than the food industry.

*“The industry also exerted its pressure through the ministries of the economic areas. So, these were multiple meetings with the authorities of these ministries [ … ] to exert pressure so that the regulation was not so harsh, to argue and counter-argue [ … ], and to present proposals to simplify the regulation, [ … ] or to make it more flexible or less drastic. [ … ] There was a lobby from the companies, and there were many presentations from companies in the National Congress”. [member of the government]*

One participant explained that food industry actors participated in the different technical meetings organised by the government and were successful in delaying these discussions.

*“[There was] an industry participation [in commissions] ( … ). And there what happened regularly was that they reached an agreement and then, at the next meeting, when it was time to read the minutes, they said that these things did not happen. So what they did was delay decision-making. In other words, the discussion of a topic could be delayed, more months and months.” [member of the government]*

*“Carozzi and Nestlé have been, perhaps, the hardest (...) They hired individuals to try to sabotage the law. They have had a very hostile attitude. Nestlé lobbied President Piñera to drop the law, along with Carozzi. (...) Piñera threw it.” [member of the government]*

The food industry, in this context, also made threats of suing the government in the Court if the Law was to be adopted.

*“The Ministry of Economy raised concerns about what was going to happen in the economic sphere. [ … ] And the direction of economic relations was very concerned about the influence that this law could have on the free trade agreements that Chile had, and about the position that the World Trade Organization could take.” [member of the government]*

*“There were many, many threats from the industry, denouncing the FOPL to the international trade law, to take it to an international tribunal. ( … ) It was a constant threat. In fact, during the discussion, it was raised that this could be unconstitutional, in an internal discussion in Chile, as it could violate the freedom of companies and enterprises.” [academic]*

*“The famous campaign of terror: “you're going to produce unemployment; if you lower the consumption of certain types of foods, the premises are going to close; I am going to have to fire workers”.” [member of the government]*

In our interviews and review of publicly available information, we found evidence that, after the adoption of the FOPL policy in 2012, food industry actors continued to exert political influence during its implementation. At the end of 2016, AB Chile launched a campaign on TV, the internet and newspapers, called “*Hagamolos bien*” (“Let’s do it right”), where celebrities questioned the policy [interview, member of the government]. While some companies have complied with the policy, others, such as Carozzi, still lobbies for changes in the policy, which causes internal tensions in the trade association AB Chile [A10 Carozzi, A30 Coca-Cola].

*“Carozzi has met with the authority to address the [FOPL policy] more than twenty times. They have talked with the sub-secretary of the Treasury [ … ]; with [ … ] the sub-secretary of Health; with the Minister of Health [ … ]; among others.” [A7, A12–4 Carozzi]*

One interviewee also noted that the sweeteners industry might have benefited from the new FOPL policy, information which was also found in the public domain: the reformulation of products to limit their level of added sugar, following the adoption of the Law, led to the increased use of sweeteners instead.

*“After the Food Labelling Law was enacted in 2016, the country's food industry began to adapt to the new parameters required by the authority. Many products that previously contained sugar, for example, were reformulated with non-caloric sweeteners.” [A52 Coca-Cola]*

Beyond that particular FOPL policy, we found other evidence of industry direct involvement in policy. For example, two of the five representatives for Chile in the 2019 Codex Alimentarius meeting for food labelling were employed by AB Chile and Coca-Cola [A4 ABChile, A65 Coca-Cola]. In July 2019, Nestlé arranged a meeting with the Sub-secretariat of Health to *“present the educational and nutritional program “Healthy Children“ that already impacts 80,000 children in Chile.”* [A90 Nestlé]. Nestlé also participated in a “*multi-sectoral event of the UN (United Nations) for Zero Hunger (...). This meeting consisted of a discussion table to work collaboratively as an engine that reverses the dramatic situation of malnutrition in Chile. ( …*) *Representatives of the FAO (Food Agriculture Organization) were present, [as well as] Nestlé, MINAGRI (Ministry of Agriculture), ( …) The University of Santiago, ( …*), *among others*.” [A88 Nestlé].

### Discursive strategies

In parallel to the instrumental strategies described above, food industry actors used a range of discursive, argument-based strategies that could have influenced public health policy, research and practices in Chile. Our interviewees for example, and as described earlier, shared details of the arguments used by the food industry when lobbying against the proposed FOPL policy: the unintended consequences that the policy would have on the economy and society; and the breaches of international trade agreements. Two years after the initial implementation of the FOPL policy, in 2019, Carozzi claimed that the policy was not successful in reaching its intended objective of addressing the issue of obesity [A7 and A21]. Carozzi stressed the fact that its sales “*on the contrary they are on the rise*” [A7], a message, which, unintentionally perhaps, recognises the fact that the products of the company contribute to the obesity epidemic.

Some participants noted that similar arguments were used, more recently, against the proposed increase on the taxation of sugar-sweetened beverages.

*“In terms of politics, the threat has always happened, both for the tax on sugary drinks and the labelling law, the threat appeared with the loss of jobs [ … ] and that this would decrease the investment of the food industry in Chile.” [academic]*

*“The reaction to [a study on taxes] was (...) that the tax doesn't solve anything, that it hurts the poor, that people are going to substitute with poorer quality foods.” [academic]*

More generally, in Chile, some industry actors presented themselves as part of the solution in controlling obesity, providing consumers with new, reformulated products, and supporting physical activity initiatives across the country (see Table 1).

*“As a company, we believe that we can and should be part of the solution to the problem of childhood obesity that Chile faces. ( …) Contributing, through our expertise in nutrition, to education for the formation of habits.” [A112 Nestlé]*

*“The good news of Coca-Cola: 95% of its products are low or calorie-free” [A45 Coca-Cola]*

*“We are convinced that the only way to defeat the scourge of obesity is through education.” [A20 Carozzi]*

*“We are interested in our consumers being healthy. ( …) For this reason, ( …) we have been present at hundreds of sports events, motivating more than a million people to join the movement.” [A20 Carozzi]*

Some food industry actors also promoted their important role, in communities and the country, in terms of environmental sustainability:

*“We are concerned with promoting water conservation to ensure the continuity of our business while contributing positively to the communities where we operate.” [A114 PepsiCo]*

Some actors in the food industry also stressed their crucial role in the economy, through the creation of employment, for example:

*“Today, with an annual investment of US $ 250 million, the group's annual economic contribution in the country is US $ 2.4 billion, which is equivalent to almost 1% of the national GDP. Likewise, across the entire value chain, it generates 103,000 direct and indirect jobs, a figure that corresponds to 1.3% of the total workforce.” [A58 Coca-Cola]*

### Global drivers and solutions to address the CPA of the food industry in Chile

Our study happened during the protests at the end of 2019 in Chile. Our interviewees noted that the CPA of the food industry, and other industries, such as the pharmaceutical and tobacco industries, was facilitated by the existence of global drivers, particularly neo-liberal policies, such as the privatization of public services (including education and healthcare), reductions in government spending, and free trade. Participants in our interviews mentioned that the population mostly accepted these neo-liberal policies and the role of corporations in the Chilean society, as these were imposed on them, until the protests started. Interviewees felt that a shift was taking place amongst Chileans: from a movement against the increase in the price of public transportation, the protests rapidly moved to question the neo-liberal system.

*“The neoliberal thinking has deeply permeated within the Chilean society, and the industry uses this very well, in its favour, within public policies.” [academic]*

*“Look how the whistles sound [n.b.: the interview took place in a neighbourhood where the protest was happening at that exact moment], because this is what society today is telling us, look it seems that this was not what that accommodated us. Because this 'let be' and ‘the market regulates itself’ (...) It seems like it was not the best strategy, after many years of having the model (...). Our model is based on the industry.” [member of the government]*

*“Until now, until the social movement, in the governments of Chile, economic concern has always been paramount. In other words, for the elite, what governs is the economy.” [member of the government]*

More specifically, our interviewees discussed potential solutions to address the CPA of the food industry, in Chile, and beyond. One member of the government, for example, noted in the interviews that the Law on Lobbying and the Law on Transparency, adopted recently in the country, *“helped a lot to control the relationship between the industry and the government. Because any meeting that the industry requested with the government had to be requested through the lobby law and it had to be publicly recorded”*. Another interviewee suggested that the Framework Convention on Tobacco Control (FCTC) could be a binding tool adapted to protect and promote healthy diets and that an independent body of the government could monitor the interactions between corporations and the government. Other mechanisms could be put in place in Chile to protect public policy from undue influence from corporations, including the public disclosure of all correspondences between the government and third parties, and the protection of whistle-blowers, for example.

One academic noted the existence of the movement “Medicos sin marcas” (‘Doctors without brands’) in Chile [29], which advocates for the practice of medicine without influence from marketing, and without conflicts of interest. This initiative may be replicated to other professions in public health and nutrition. Two interviewees suggested that an independent pool of funds for research could be created in the country, for which industry could contribute but not decide on the distribution of funding. Two other interviewees noted that scientific conferences could be held independently, without sponsorship from the industry, which is already the case for the annual Congress of the Chilean Society of Family Medicine [30], for example. Our interviewees noted that events could be held in venues like universities and could use video conferences.

## Discussion

In Chile, we found 130 examples of CPA used by the food industry during data collection through our document analysis. Some of this data was triangulated with data from our interviews, and our interviewees shared additional examples of CPA. In Chile, food industry actors supported different community initiatives, particularly those targeted at children and those focusing on environmental sustainability. These initiatives sometimes exposed their recipients to the brands of the companies, with the visits of factories and/or the distribution of products, amongst other vehicles of promotion. This was also documented in previous studies in countries such as Brazil, Ecuador or Fiji, amongst others [9, 22, 26]. In Chile, environmental sustainability initiatives and those targeted at the control of obesity were common. We also found evidence that food industry actors, such as Nestlé, funded research prizes and scholarships to researchers, for example. We identified eights scientific events in 2019 sponsored by food industry actors, principally Nestlé and ILSI. Similar practices were identified in other studies of the CPA of the food industry [26], including in France [31] and Australia [24]. This influence of the food industry on research risks disseminating biased information to policymakers and the public more generally, as well as compromising the independence and integrity of researchers and research institutions in the country [26]. In Chile, food industry actors, including those represented by former members of the government, lobbied against the development and implementation of the FOPL policy. Lobbying is a common practice of the food industry, as noted in previous studies [22, 24, 26, 32]. The Ministries of Economy, Agriculture and Foreign Affairs also supported the industry position. When lobbying against public health policies, food industry actors claimed that there would be unintended negative consequences for society and the economy and that the proposed policies would breach trade agreements. Similar arguments were for example used in Fiji against the development of public health policies [22]. Since the adoption of the FOPL Law 20.606, there are internal conflicts in the industry between those who support it and those who still criticised and want to modify it. Finally, in their arguments used in public and private spaces, we found that food industry actors stressed their crucial role in the Chilean economy. They presented themselves as part of the solution in the prevention and control of obesity, with a particular focus on their efforts to reformulate their products and their support to physical activity initiatives across the country. These strategies, however, lack evidence of their effectiveness in improving population health and, on some occasions, could be used to undermine efforts to promote healthy diets and prevent and control obesity [33]. Particularly worrying for some interviewees is the increased use of sweeteners after the implementation of the FOPL policy. The impact of that policy on population health, beyond the reduction of certain nutrients and calories in the market, needs to be explored, as the level of processing of food products may have remained identical before and after its implementation [34].

In the context of the protests, the neo-liberal system was questioned by the social movement. Chile is indeed known internationally for its neo-liberal system, where the State has little interference with the market-driven economy [35]. This system was implemented in the 1970s, during the Pinochet dictatorship period, on the advice of the Chicago Boys, returning from their training in the USA with the famous economist Milton Friedman [35]. The neo-liberal system now drives the global economy. In 2019, these protests in Chile had parallels in other countries like Lebanon [36] and France [37], and adds to the existing criticism of the neo-liberal system made by movements like Occupy Wall Street [38] or the ‘Global Campaign to Reclaim Peoples Sovereignty, Dismantle Corporate Power and Stop Impunity’, made of more than 250 groups affected by the activities of corporations [39]. A referendum with a proposal to change the Constitution is planned to be held in Chile late 2020 [40] and could have an impact on the political practices described in the present publication.

Specific solutions to address the CPA of the food industry, such as increased transparency in the policy process and the protection of scientific independence, were also discussed by our interviewees. Given the recent coronavirus outbreak, more scientific events around the globe are already held online. This could be perhaps be sustained in the longer term. Online events could: i) reduce industry influence on science; ii) reduce inequalities, for early career researchers and those from low-income countries (who do not have access to these events due to limited funding for example), for academics who serve as caregivers to a family member, and for mothers who breastfeed (and would then not need to separate from their children), for example; iii) reduce our negative impact on the environment, with the decrease of business trips. Another suggestion was the introduction of a binding tool equivalent to the FCTC, which has been called for internationally as well [21]. Importantly, the current COVID-19 outbreak could be used by commercial enterprises, in Chile and beyond, to further advance their economic interests, even if those were questioned during the social protests. For example, the alcohol industry has been shown to push for the categorisation of alcohol as an essential commodity during the lockdowns [41], despite the negative impacts of alcohol drinking on health.

Our study has some limitations. First, due to a large amount of data available online, it was impossible to search at the declarations of interests of all members of the government. This could be the subject of a specific study. In addition, due to time constraints, we could not focus on certain actors of the food industry: the breast-milk substitutes industry, the sweeteners industry, the dietary supplements industry, the SOFOFA (the trade association of all industries in Chile). More research is therefore needed for these actors. Moreover, and as detailed in our methodology section, several food industry actors did not have a national website and/or social media account. Importantly, we neither studied the rationale and context for these practices to be used by the industry, nor did we study the impact they had on policy. This should be the subject of further analyses. In addition, we may have found more examples of CPA practices for some industry actors and less for others, but this must not be interpreted as an actor having more influence. Indeed, some actors may use these practices “behind closed doors”, and we did not have access to that information. In that case, using other sources of information, such as interviews or internal documents, could help in retrieving relevant information, but certain forms of influence, such as dinner invitations or phone calls, will remain complicated to discover.

Our findings are consistent with research conducted on the CPA of the food industry in other countries [9, 22–25, 32], as noted above, as well as with evidence of the CPA of the alcohol and tobacco industries [15, 42, 43]. Of particular relevance is the inclusion of food industry actors that are part of the International Food and Beverage Alliance. Studying the differences and similarities in the behaviours of these companies across countries might be the subject of future studies. A difference in the case of Chile is the support for environmental sustainability initiatives, in addition to those for obesity control and physical activity. In addition, the lobby against public health policies, in the Congress and on the Executive branch of the government, was not only directly exerted by food industry actors, but also by former members of the government (using the ‘revolving door’), and through other Ministries, who repeated the arguments of the industry.

## Conclusion

In Chile, evidence from the public domain and data collected through interviews revealed that the food industry used action- and argument-based CPA practices which could influence public health policy, research and practice, including building alliances with communities and in academia. While the country has some measures in place to manage the interactions between the industry, and the government and public health professionals, there is still a need to develop robust mechanisms to address undue influence from corporations.

## Supplementary Information

**Additional file 1.** Conceptual framework for categorising the corporate political activity of the food industry.

**Additional file 2.** Sources of information to identify the corporate political of the food industry in Chile.

**Additional file 3.** Data collected from publicly available information.

**Additional file 4.** Interview guide (Spanish).

**Additional file 5.** Conceptual framework for categorising the corporate political activity of the food industry.

## Data Availability

All data from the public domain collected during this study are available with this manuscript as Additional file 3. Data from our interviews are available from the corresponding author on reasonable request.
