# Supplementary material for: Food industry political practices in Chile: “the economy has always been the main concern”
Source: Global Health. 2020 Oct 27;16:107. doi: 10.1186/s12992-020-00638-4 (PMC7590241; doi:10.1186/s12992-020-00638-4)
Supplement: Supplementary file 2 — Additional file 2. Sources of information to identify the corporate political of the food industry in Chile. [file 12992_2020_638_MOESM2_ESM.docx]

App 2: Sources of information consulted for the study of the CPA of the food industry in Chile in 2019

| **Nature of the source of information** | **Source of information** | **Country-specific URL** |
| --- | --- | --- |
| Industry websites | Country-specific website of the industry actor | o Coca Cola:  https://www.cocacoladechile.cl/  http://www.koandina.com/ - need to study annual report in more details in future studies (sustainibility etc): http://www.koandina.com/uploads/Memoria%20Anual%20Integrada%202018.pdf http://www.cocacolalatinamerica.com/es/cl/home/ - only commercial - promotion http://www.embonor.cl/wps/wcm/connect/embonor/Sitio/Home  o Danone: bought out by Watt's in 2017, not included o Ferrero/AgriChile: https://www.agrichile.cl/agrichile no relevant information o General Mills: no national website  o Grupo Bimbo/Ideal: no national website  o Kellogg’s: http://www.kelloggs.cl/es_CL/home.html - not working during data collection o Mars: no national website o McDonald’s: http://www.mcdonalds.cl/  o Mondelez: no national website o Nestlé:  https://www.nestle.cl/  https://www.nestle-contigo.cl/ - Multiple webpages on nutrition and physical activity - need to do a case study in future studies o PepsiCo: http://www.pepsico.cl/home  http://www.pepsicocomunidad.cl/  o Unilever: https://www.unilever.cl/ - not data specifically for Chile - all about Unilever global  o Carozzi Alimentos: https://www.carozzicorp.com/  o PF Alimentos: https://www.pfalimentos.cl/ Not updated since 2016 o Soprole: https://www.soprole.cl/es.html   o Asociación Chilena de Ciencia y Tecnología en Alimentos (Sochital): http://sochital.com/ - not working during data collection  o ILSI: http://www.ilsisurandino.cl/sitio/ not much relevant information, much of the website not updated since 2014-5 o ChileAlimentos: http://www.chilealimentos.com/wordpress/  o Asociación Gremial De Alimentos Y Bebidas De Chile A.G. - AB Chile: http://www.abchile.cl/  o Supermercados de Chile A.G.: https://www.supermercadosdechile.cl/ - no relevant information  o Campaign “Hagamos lo bien”: https://es-la.facebook.com/hagamoslobien.cl/ - not included because last post was in 2016 |
| Government websites | Websites of Ministry in charge of health and related agencies (National level), Ministry of Education | Instituto de Salud Pública de Chile: http://www.ispch.cl/ - no relevant info - all on the Transparency portal Ministerio de Salud: http://www.minsal.cl/ - no relevant info  Ministerio de Educación: https://www.mineduc.cl/ - no relevant info |
|  |  |  |
|  |  |  |
|  | Websites of the Parliament and Senate (National level) | Gobierno Transparente: https://transparenciaactiva.presidencia.cl - no relevant info - all on the Transparency portal Senado: http://www.senado.cl - - no relevant info - all on the Transparency portal Camara de Diputados: http://www.camara.cl - no relevant info - all on the Transparency portal |
|  |  |  |
|  |  |  |
|  | Other | **Relevant websites of the Consejo para la Transparencia CPLT:**  **Info Probidad**: Contiene los conjuntos de datos de las Declaraciones de Intereses y Patrimonio de los sujetos obligados de la Ley N°20.880 sobre probidad en la función pública y prevención de los conflictos de intereses - https://www.infoprobidad.cl/#!/inicio NOT all , too much information, need a separate study  **Info Lobby:** Audiencias de Lobby, Viajes y Donativos de las autoridades del Estado de Chile - https://www.infolobby.cl/#!/busqueda-simple - could do another study of the relationships between the lobbyists of different companies  **Datos Abiertos :**catálogo de datos abiertos (opendata) del Consejo para la Transparencia es una iniciativa piloto en OpenData. Dispone de datos en bruto - https://www.consejotransparencia.cl/datos-abiertos/ NOT USED, no information for 2019  **Plataforma Ley de Lobby**: public register of lobbyists: https://www.leylobby.gob.cl/lobbistas?it=%2Flobbistas&page=6 Consulted Info Lobby |
|  | Websites of major political parties and websites of commissions in charge of elections (National and State Level) | Unión Demócrata Independiente: http://www.udi.cl/ - not workinf during data collection Renovación Nacional: www.rn.cl - no relevant information Partido Socialista de Chile: www.pschile.cl - a webpage on transparency with much information, no data on the CPA of the food industry Partido Demócrata Cristiano: www.pdc.cl - a webpage on transparency with much information, no data on the CPA of the food industry Partido por la Democracia: www.ppd.cl |
| Other material | Websites of major universities with a School/Department of nutrition/dietetics/exercise or physical activity | Universidad de Chile: http://www.uchile.cl  INTA: https://inta.cl/  Universidad de Chile: http://www.uchile.cl  Universidad de Valparaiso: http://www.uv.cl - - no relevant information  Universidad de Concepcion: http://www.udec.cl/pexterno/ - no relevant information  Universidad de la Frontera: https://www.ufro.cl - no relevant information  Universidad del Bio-Bio: http://webfacsa.ubiobio.cl/ - no relevant information |
|  |  |  |
|  | Websites of major conferences on diet-, public health- or physical activity-related issues (National level) | XXII Congreso Chileno de Obesidad (SOCHOB) 2019 - no info online  XXII Congreso Nacional de Nutrición (SOCHINUT) 2018: https://cdb4224e-93a8-47c8-8331-ee33343c6d74.filesusr.com/ugd/a9223b_64460dab789b461ca8e031841870b806.pdf - not much info and no evidence of CPA  X Congreso Chileno de Nutrición Clínica, Obesidad y Metabolismo, III Congreso de Nutrición Clínica Pediatrica (ACHINUTMET) 2019: http://www.achinumet.cl/Congreso-2019/ - also presence of pharma industry but not covered here  Colegio de Nutricionistas de Chile A.G - https://www.colegiodenutricionistas.cl/congreso2019  XXX Congreso Chileno de Endocrinología y Diabetes (SOCHED) 2019: http://soched.cl/web/bienvenida-2019/ - , only pharma industry |
|  |  |  |
|  |  |  |
|  |  |  |
|  | Websites of major professional bodies in diet-, public health- or physical activity related health issues (National level) | Sociedad Chilena de Obesidad (SOCHOB): http://www.sochob.cl/  Sociedad Chilena de Nutrición (SOCHINUT): https://www.sochinut.cl  Asociación Chilena de Nutrición Clínica, Obesidad y Metabolismo (ACHINUMET): http://www.achinumet.cl  Colegio de Nutricionistas Universitarios de Chile A.G: https://www.colegiodenutricionistas.cl/  Sociedad Chilena de Endocrinología y Diabetes (SOCHED): http://soched.cl/web/ |
|  |  |  |
|  |  |  |
| News | News and media releases | **Newspapers:** El Mercurio: emol.com - Not freely accessible  La Tercera: latercera.com - Not all articles were freely accessible  La Segunda: lasegunda.cl - Not freely accessible |
| Industry social media | Twitter account - national only | Bimbo - Ideal: https://twitter.com/bimboideal - no update in 2019  Coca Cola: https://twitter.com/cocacolacocl - mostly about sustainibility https://twitter.com/cocacola_cl - only until July 2019, not many tweets https://twitter.com/cocacolaformecl - just about promotion  Mc Donald´s: https://twitter.com/mcdonalds_cl - just about promotion  Nestle:  https://twitter.com/nestlecl -  https://twitter.com/savorychile - no update in 2019 https://twitter.com/nestlefitnesscl - no update in 2019  PepsiCo: https://twitter.com/PepsiCoChile -   Soprole: https://twitter.com/soprole - no update in 2019 PF Alimentos: https://twitter.com/senor_pf - only until July 2019, just about promotion Carozzi: https://twitter.com/CarozziHablemos - only until July 2019 - only 2 messages in 2019  ILSI Sur Andino: https://twitter.com/ilsisurandino -   ChileAlimentos: https://twitter.com/chilealimentos - , mostly about business |
