# Supplementary material for: Food industry political practices in Chile: “the economy has always been the main concern”
Source: Global Health. 2020 Oct 27;16:107. doi: 10.1186/s12992-020-00638-4 (PMC7590241; doi:10.1186/s12992-020-00638-4)
Supplement: Supplementary file 3 — Additional file 3. Data collected from publicly available information. [file 12992_2020_638_MOESM3_ESM.docx]

| **Code** | **Actor** | **Source** | **Strategy** | **Code** | **Further details for codes (when applicable)** | **Data coded** | **Notes** | **Website URL** | **Date collected** |
| --- | --- | --- | --- | --- | --- | --- | --- | --- | --- |
| A1 | AB Chile | Government | Direct involvement and influence in policy | Lobby |  | Francisca Aguirre Boza SUBSECRETARÍA DE SALUD PÚBLICA  Jefa (S) de Departamento de Alimentos y Nutrición - DIPOL SSP  Fecha de Inicio: 22 de agosto de 2019  Observación: Temas a tratar: Se solicita audiencia con el Sr. Ministro Jaime Mañalich para saludo protocolar. Temas tratados. Como se expresan las porciones, los diferentes productos. Concepto de variabilidad +- 30 Variabilidad aceptada concepto de envase individual solicita conocer nuestra opinión y solicitan se les indique cual es la correcta forma de expresar loas medidas casera y porciones Se les responde de que será analizado por el equipo técnico  Materia: Diseño, implementación y evaluación de políticas, planes y programas efectuados por los sujetos pasivos. -  Lugar Audiencia: Santiago  Datos Principales 22 de agosto de 2019  Región Región Metropolitana de Santiago  Tipo de Registro Audiencia ASOCIACIÓN GREMIAL DE ALIMENTOS Y BEBIDAS DE CHILE A.G. |  | [https://www.infolobby.cl/app/src/index.html#!/busqueda-avanzada/eb1c1db4-5158-4e0d-b727-58909535f907](https://www.infolobby.cl/app/src/index.html#!/busqueda-avanzada/eb1c1db4-5158-4e0d-b727-58909535f907) | 26-Dec-19 |
| A2 | AB Chile | Website industry | Information management | Amplification |  | “Edulcorantes bajos en calorías en el debate actual de salud pública” Organizado por la Asociación Internacional de Edulcorantes (ISA) se llevó a cabo el 14 de agosto de 2019 en Santiago de Chile.  pueden acceder al video que se presentó sobre el Comité Mixto FAO/OMS de Expertos en Aditivos Alimentarios (JECFA) aquí:  https://youtu.be/GsijNEiEvnI |  | <http://www.abchile.cl/index.php?mact=News,cntnt01,detail,0&cntnt01articleid=690&cntnt01returnid=59> | 31-Dec-19 |
| A3 | AB Chile | Website industry | Information management | Amplification |  | jul 11, 2019 Fuente: El Mercurio Categoría: Internacionales El café puede ser la clave contra la obesidad y la diabetes Científicos de la Universidad de Nottingham, en Inglaterra, han descubierto que tomar una taza de café al día puede estimular la llamada grasa parda, un tipo de tejido adiposo que quema energía para producir calor y eliminar la acumulación de grasa blanca, característica de la obesidad. | Other examples: http://www.abchile.cl/index.php?mact=News,cntnt01,detail,0&cntnt01articleid=678&cntnt01detailtemplate=ab-detailnews&cntnt01returnid=59 http://www.abchile.cl/index.php?mact=News,cntnt01,detail,0&cntnt01articleid=676&cntnt01detailtemplate=ab-detailnews&cntnt01returnid=59 http://www.abchile.cl/index.php?mact=News,cntnt01,detail,0&cntnt01articleid=639&cntnt01detailtemplate=ab-detailnews&cntnt01returnid=59 http://www.abchile.cl/index.php?mact=News,cntnt01,detail,0&cntnt01articleid=638&cntnt01detailtemplate=ab-detailnews&cntnt01returnid=59 | <http://www.abchile.cl/index.php?mact=News,cntnt01,detail,0&cntnt01articleid=679&cntnt01detailtemplate=ab-detailnews&cntnt01returnid=59> | 31-Dec-19 |
| A4 | AB Chile | Other | Direct involvement and influence in policy | Actors |  | PROGRAMA CONJUNTO FAO/OMS SOBRE NORMAS ALIMENTARIAS COMISIÓN DEL CODEX ALIMENTARIUS 42.o período de sesiones Ginebra (Suiza) 8–12 de julio de 2019 INFORME DE LA 45.a REUNIÓN DEL COMITÉ DEL CODEX SOBRE ETIQUETADO DE LOS ALIMENTOS Ottawa (Ontario, Canadá) 13–17 de mayo de 2019 REP19/FL Appendix I 21 Appendix I LISTA DE PARTICIPANTES Mrs Marisol Figueroa Barrientos Gerente General y Gerente Técnico de Alimentos y Bebidas de Chile A.G. (AB Chile) Alimentos y Bebidas de Chile A.G. - AB Chile Los Militares 6191, oficina 71, piso 7, Las Condes. Santiago Chile Email: mfigueroa@abchile.com Mrs Gisela Rodríguez Rideau Gerente Asuntos Científicos y Regulatorios Coca-Cola Avenida Kennedy 5757, piso 12. Las Condes Santiago Chile Tel: +56 2 233834209 Email: gisrodriguez@coca-cola.com |  | <http://www.fao.org/fao-who-codexalimentarius/sh-proxy/en/?lnk=1&url=https%253A%252F%252Fworkspace.fao.org%252Fsites%252Fcodex%252FMeetings%252FCX-714-45%252FFinal%252520Report%252FREP19_FLs.pdf> | 01-Nov-19 |
| A5 | Carozzi | Media | Direct involvement and influence in policy | Indirect access | Lobby | En marzo, AB Chile redefinirá su postura respecto a la Ley de Etiquetado. Ese mes, el presidente del gremio, Rodrigo Álvarez, le propondrá al directorio un nuevo “rayado de cancha” respecto a la normativa. Es que las diferencias entre dos de sus socios han derivado en la necesidad de coordinar otro discurso común. Las divergencias entre Coca-Cola y Carozzi han permeado a la institución. El choque entre ambos actores -donde, en líneas generales, uno respalda la normativa, y el otro quiere profundas modificaciones- ha hecho que incluso existan peticiones de sanción sobre uno de ellos, mientras el otro evalúa permanentemente dejar el gremio. En el intertanto, ya se alista una reforma a los estatutos y un código de ética que los norme. (...) Todo, para lograr aunar posiciones en una soterrada disputa que ya suma varios años y capítulos, con -por un lado- un Carozzi desarrollando un intenso lobby para modificar la ley en pos -dicen- de combatir realmente la obesidad; y -por otro- un Coca-Cola valorando la normativa, y avanzando -como han repetido- a pasos agigantados en reducir los niveles de azúcar, y por consiguiente, en eliminar los sellos de sus productos. “Ambas empresas han estado en algún momento peleadas con todas las demás. |  | <https://www.latercera.com/pulso/noticia/la-disputa-carozzi-coca-cola-impulsa-una-nueva-definicion-gremial-frente-etiquetado/522607/> | 22-Nov-19 |
| A6 | Carozzi | Media | Discursive | Frame the debate | Part of the solution | Desde Carozzi aseguran que todas las empresas de AB están trabajando para lograr controlar la obesidad, proponiendo iniciativas para que las personas tengan acceso a más y mejor información. |  | <https://www.latercera.com/pulso/noticia/la-disputa-carozzi-coca-cola-impulsa-una-nueva-definicion-gremial-frente-etiquetado/522607/> | 22-Nov-19 |
| A7 | Carozzi | Media | Direct involvement and influence in policy Discursive | Indirect access Intended public health benefits | Lobby | Más de una veintena de veces se ha reunido Carozzi con la autoridad para abordar la Ley de Etiquetado. Han conversado con el subsecretario de Hacienda, Francisco Moreno; con Daniela Godoy, de la subsecretaria de Salud; con el ministro de Salud, Emilio Santelices; con Felipe Lopeandía, de la Direcon, entre otros. Si bien al interior de la firma descartan hacer lobby, la lucha por modificar la normativa -y que en vez de 100 gramos, se mida en porciones de consumo- se ha transformada en una disputa personal. Es que es el mismo dueño y presidente de la firma, Gonzalo Bofill, quien ha asumido ese desafío. Para ellos, el interés radica en que la normativa no combate la obesidad, cuestión fundamental para tener consumidores sanos. De hecho, sus ventas no han caído, por el contrario van en alza. En el sector, precisan no obstante, que el temor está en que en un futuro -una vez acreditado que la norma no redujo la obesidad- se intensifiquen las medias contra la industria. (...) El 17 de diciembre, Gonzalo Bofill llegó a las oficinas del ministro Santelices. ¿El motivo? Explicarle una nueva propuesta: incorporarle información adicional a los sellos en relación a la porción a consumir. |  | <https://www.latercera.com/pulso/noticia/la-disputa-carozzi-coca-cola-impulsa-una-nueva-definicion-gremial-frente-etiquetado/522607/> | 22-Nov-19 |
| A8 | Carozzi | Media | Information management | Amplification |  | Tal ha sido [la] arremetida [de Carozzi], que han encargado estudios al respecto y contrataron una agencia de comunicaciones sólo para ese tema, B2o. Más aún, diseñaron una propuesta en solitario para presentarle a la autoridad. El último dolor de cabeza al interior de AB. |  | <https://www.latercera.com/pulso/noticia/la-disputa-carozzi-coca-cola-impulsa-una-nueva-definicion-gremial-frente-etiquetado/522607/> | 22-Nov-19 |
| A9 | Carozzi | Media | Discursive | Frame the debate | Part of the solution | En [su nueva propuesta] dejaban claro no oponerse a la ley, sino que ayudar a mejorarla para combatir realmente la obesidad. |  | <https://www.latercera.com/pulso/noticia/la-disputa-carozzi-coca-cola-impulsa-una-nueva-definicion-gremial-frente-etiquetado/522607/> | 22-Nov-19 |
| A10 | Carozzi | Media |  |  | Negative case - the industry does not agree with the approach taken by Carozzi | Y de nuevo hubo ruido: Carozzi nunca socializó tal iniciativa con AB Chile, nunca la expuso, los socios prácticamente se enteraron por lo medios. Y nuevamente se habrían pedido sanciones sobre la compañía. Incluso Coca-Cola evaluó nuevamente retirarse; un hecho que aún no se descarta. “Hay un cierto cansancio o una cierta convicción de que no tiene sentido discutir lo del sello”, dicen conocedores. Añaden que Carozzi sigue insistiendo en agregarle datos al mismo disco pare, cuando se puede avanzar en algo similar a lo que hace Coca-Cola o Nestlé, poniendo pilas con la cantidad de calorías y cuánto representa de la ingesta diaria, o las porciones sugeridas al lado de los sellos. Es que en eso sí hay unidad: se debe avanzar en más información, pero para muchos la estrategia de Carozzi ya está agotada. De hecho, altas fuentes al interior de la misma compañía sostienen que las autoridades ya no los escuchan. Para algunos, por falta de una preocupación real ante la obesidad, para otros, por cansancio. Lo que sí está claro es que en marzo todos los directores de AB Chile se verán nuevamente las caras. Y escucharán a Rodrigo Álvarez. Revisión de estatutos, un código de ética y nuevos lineamientos para afrontar la Ley de Etiquetado serán parte de la tabla. Es que una disputa que partió de a dos hoy exige nuevas definiciones a nivel gremial. Aún les quedan muchos temas que afrontar, por lo que al final del día es más conveniente seguir unidos. |  | <https://www.latercera.com/pulso/noticia/la-disputa-carozzi-coca-cola-impulsa-una-nueva-definicion-gremial-frente-etiquetado/522607/> | 22-Nov-19 |
| A11 | Carozzi | Media | Coalition management Discursive | Community Frame the debate | Physical activity | Los jóvenes y los niños mostraron todo su temple en el Carozzi Kids Challenge.  (...) Los casi cero grados de temperatura que había esta mañana a las 07:45 horas para el inicio del “Carozzi Kids Challenge” de Pucón no fue impedimento para que con todo entusiasmo y vigor 600 jóvenes y niños participaran de esta prueba donde se forjan los futuros grandes triatletas chilenos.  La competencia, que reunió a 600 triatletas, se dividió en seis categorías donde fueron entrando al agua las series mayores y finalmente, cuando ya el sol comenzó a temperar la playa, ingresaron las series menores |  | <https://www.latercera.com/nacional/noticia/los-jovenes-los-ninos-mostraron-temple-carozzi-kids-challenge/482427/> | 22-Nov-19 |
| A12 | Carozzi | Government | Direct involvement and influence in policy Information management Discursive | Lobby Amplification Frame the debate | Part of the solution | Audiencia Datos de Audiencia Organismo público que informa: Subsecretaría De Salud Pública  Duración: 60 minuto(s)  Lugar: Santiago  Fecha Audiencia: 2 de julio de 2019 11:05 Observaciones: TEMAS A TRATAR: Presentación de propuesta de perfeccionamiento del etiquetado de alimentos y estudio de percepción pública sobre esta propuesta. TEMAS TRATADOS: Representantes de empresa carozzi proponen al MINSAL un cambio en los sellos "alto en". Muestran presentación con la propuesta y respaldo por estudio CADEM. Se conversa sobre resultados de la propuesta .) del Depto. de Nutrición y alimentos le es de interés escuchar propuestas de mejora de la Ley. 2)De la propuesta se les menciona limitaciones metodológicas importantes del estudio. 3) Se esperará oficio para entregar respuesta formal a su propuesta. El Depto. de Nutrición y Alimentos enfatiza en el error de la afirmación que "la Ley no ha tenido buenos resultados al mirar las cifras de obesidad", y se les aclara que no es el objetivo de la ley. se les anima a conocer de la ley 20.606 en pagina web de MINSAL  Nombre: Francisca Aguirre Boza  Lobbistas o Gestores de Interés  Nombre: Santiago Valdes  Calidad: Gestor Nacionalidad: Chile Trabaja para: Empresas Carozzi S.A.  Persona o Entidad a la que Representa: Empresas Carozzi S.A.   Nombre: Luz María Rojas  Calidad: Gestor Nacionalidad: Chile Trabaja para: Empresas Carozzi S.A.  Persona o Entidad a la que Representa: Empresas Carozzi S.A. |  | <https://www.infolobby.cl/Ficha/Audiencia/ao0013603441> | 26-Dec-19 |
| A13 | Carozzi | Government | Direct involvement and influence in policy Information management Discursive | Lobby Amplification Frame the debate | Part of the solution | Audiencia Datos de Audiencia Organismo público que informa: Subsecretaria De Servicios Sociales  Duración: 30 minuto(s)  Lugar: Santiago  Fecha Audiencia: 31 de mayo de 2019 15:00 Observaciones: Presentar estudio CADEM propuesta sellos de advertencia  Datos Sujeto Pasivo  Nombre: María Daniela Godoy Gabler  Lobbistas o Gestores de Interés  Nombre: Santiago Valdes  Calidad: Gestor Nacionalidad: Chile Trabaja para: Empresas Carozzi S.A.  Persona o Entidad a la que Representa: Empresas Carozzi S.A.   Nombre: Luz María Rojas  Calidad: Gestor Nacionalidad: Chile Trabaja para: Empresas Carozzi S.A.  Persona o Entidad a la que Representa: Empresas Carozzi S.A. |  | <https://www.infolobby.cl/Ficha/Audiencia/ai0083501901> | 26-Dec-19 |
| A14 | Carozzi | Government | Direct involvement and influence in policy Discursive | Lobby Frame the debate | Part of the solution | Audiencia Datos de Audiencia Organismo público que informa: Subsecretaria De Evaluación Social  Duración: 60 minuto(s)  Lugar: Santiago  Fecha Audiencia: 31 de enero de 2019 10:00 Observaciones: Durante la reunión se abordó propuesta de la empresa para contribuir a políticas públicas que entreguen más y mejor información a las personas y promuevan hábitos saludable, de manera de aportar al desarrollo social del país, presentación del trabajo que realiza la empresa en perfeccionar sus alimentos (17 categorías), proponer que se entregue mayor información sobre los alimentos que se compren para que así la población tome mejores decisiones.  Datos Sujeto Pasivo  Nombre: Alfredo Moreno Charme Cargo: Ministro  Lobbistas o Gestores de Interés  Nombre: Luz María Rojas  Calidad: Gestor Nacionalidad: Chile Trabaja para: Empresas Carozzi S.A.  Persona o Entidad a la que Representa: Empresas Carozzi S.A.   Nombre: Sebastian Garcia  Calidad: Gestor Nacionalidad: Chile Persona o Entidad a la que Representa: Empresas Carozzi S.A. |  | <https://www.infolobby.cl/Ficha/Audiencia/ai0073199231> | 26-Dec-19 |
| A15 | Carozzi | Twitter | Discursive | Frame the debate | Part of the solution Personal responsibility | Carozzi Hablemos @CarozziHablemos Suivre Suivre @CarozziHablemos Porque en Carozzi estamos comprometidos con la salud de nuestros colaboradores, los motivamos y ayudamos a que tengan un estilo de vida más saludable. Revisa más detalles de #Movimiento5x30 y nuestras iniciativas en 👉 https://bit.ly/30USfPP y súmate a #Carozzi5x30 0:12 16:46 - 23 juil. 2019 |  | <https://twitter.com/CarozziHablemos/status/1153813594145902592> | 27-Dec-19 |
| A16 | Carozzi | Twitter | Discursive | Frame the debate | Part of the solution Personal responsibility | Carozzi Hablemos @CarozziHablemos Suivre Suivre @CarozziHablemos En Carozzi estamos comprometidos con el bienestar de las familias, por eso fomentamos el deporte y la actividad física para combatir el sedentarismo. Conoce más de nuestras iniciativas haciendo click en 👉http://bit.ly/2SoK1wg y súmate a #Carozzi5x30 #Movimiento5x30 0:14 15:17 - 19 juil. 2019 |  | <https://twitter.com/CarozziHablemos/status/1152341644257218562> | 27-Dec-19 |
| A17 | Carozzi | Website industry | Coalition management | Community |  | Carozzi y Artequin Viña del Mar lanzan un recetario para niños  (…) Impresa en Valparaíso, la publicación de 1.000 ejemplares fue financiada con aportes de Empresas Carozzi, compañía de consumo masivo que financia por segunda vez una publicación del museo. |  | <https://www.carozzicorp.com/noticia/carozzi-y-artequin-vina-del-mar-lanzan-un-recetario-para-ninos/> | 31-Dec-19 |
| A18 | Carozzi | Website industry | Coalition management Discursive | Community Frame the debate | Physical activity Personal responsibility Part of the solution | Plan AM: alimentación y movimiento para una vida más saludable En Carozzi tenemos un tremendo desafío: promover el deporte y una alimentación equilibrada para un estilo de vida saludable. ¿En qué consiste nuestro Plan AM? ¡Te invitamos a conocerlo! 1.Jornadas AM Público objetivo: Universidades Medio: Charla técnicas en Carozzi Nos Impacto: 2000 estudiantes La actividad -dirigida a alumnos de educación superior de las áreas de la salud y la educación- incluye una visita de los estudiantes a nuestro Centro Productivo de Nos. Allí disfrutan una charla preparada especialmente por el INTA sobre la importancia de comer todos los grupos de alimentos en porciones adecuadas, manteniendo siempre horarios establecidos y una vida en movimiento. Luego visitan la Planta de Galletas y el Laboratorio de la compañía para conocer de la elaboración de nuestros productos.  2. Charlas AM Público objetivo: Apoderados y profesores Medio: Charla técnica en establecimientos educacionales Impacto: 1000 profesores y apoderados. Carozzi ha extendido las Jornadas AM –que hasta el año pasado solo se habían realizado a alumnos de universidades– a los padres y apoderados de varios colegios de nuestro país. El objetivo es traspasar a través de una charla nutricional información práctica sobre alimentación, porciones adecuadas y estilo de vida saludable y activo enfocado en la cotidianidad, con actividades tan comunes como ayudar en las tareas del hogar, salir en familia, pasear a la mascota y ejercitarse tres veces a la semana.  3. Carozzi Tour de la Energía Público objetivo: Niños Medio: Carozzi Tour de la Energía Itinerante Impacto: 7.000 niños El Carozzi Tour de la Energía es una iniciativa que busca acercar a los colegios nuestros procesos productivos de manera entretenida, educando sobre la importancia del consumo en porciones y gasto energético. La carpa inflable -un símil de nuestra planta de pastas de Nos- supone toda una experiencia interactiva. Los niños utilizan lentes de realidad virtual que les permiten conocer a través de una tecnología 360º, la preparación de productos como nuestro Cereal Bar. También se enfrentan a un escáner que indica cuáles son las porciones que se deben consumir durante el día además de participar en sopas de letras deportivas. | See pictures: branding  See also: https://www.carozzicorp.com/noticia/jornadas-am-para-padres-un-enfoque-de-alimentacion-y-movimiento-en-familia/ | <https://www.carozzicorp.com/noticia/plan-am-alimentacion-y-movimiento-para-una-vida-mas-saludable/> | 31-Dec-19 |
| A19 | Carozzi | Website industry | Coalition management Discursive | Community Frame the debate | Physical activity Personal responsibility Part of the solution | Alimentarnos equilibradamente, con un menú variado, rico en frutas, vegetales, carbohidratos, grasas y proteínas nos ayuda a obtener diariamente todos los nutrientes que nuestro organismo necesita. Si esto se transforma en un hábito, cuidaremos nuestra salud y aseguraremos efectos positivos a largo plazo.  8 consejos básicos  1.Ordena tu día en base a 3 o 4 comidas diarias + 1 colación consumiendo cada 3 o 4 horas porciones equilibradas para ayudar a tu metabolismo y entregar a tu cuerpo la energía necesaria para tu día.  2.Incorpora frutas y verduras. Son una buena fuente de vitaminas, minerales y fibra que ayudan a cuidar tu salud. La recomendación: consume 2 a 3 porciones de frutas al día y 2 a 3 porciones de verduras al día.  3.Prefiere aceites en crudo (3 a 4 cucharaditas al día) para agregar a tus comidas o ensaladas.  4.Prefiere preparaciones al horno, a la plancha o cocidas.  5.Prefiere el consumo de pescado, pollo y pavo.  6.Prefiere el consumo de lácteos descremados.  7.Hidrátate durante el día, consume entre 6-8 vasos de agua para mantener las funciones normales de tu cuerpo.  8.Realiza deporte semanalmente. Te recomendamos ejercicio moderado 5 veces a la semana por un mínimo 30 minutos #Carozzi5x30. | Several other blog posts to describe these advice - not all collected here | <https://www.carozzicorp.com/post/como-es-un-menu-balanceado/> | 31-Dec-19 |
| A20 | Carozzi | Website industry | Coalition management Discursive | Community Frame the debate | Education Personal responsibility Part of the solution | Estamos comprometidos con el bienestar de la sociedad y nos hacemos cargo de la responsabilidad que, como empresa productora de alimentos, nos cabe en la alimentación y la salud de nuestros consumidores. Estamos convencidos que la única forma de derrotar el flagelo de la obesidad, es por medio de la educación. (...) Reconocemos el mérito de la Ley de Alimentos de generar conciencia transversal sobre la importancia de controlar el consumo de nutrientes críticos, como son el sodio, el azúcar y las grasas saturadas.(...)  A través de la educación, tanto de las porciones adecuadas de consumo como del contenido nutricional de cada producto, queremos que nuestros consumidores sepan qué están realmente comiendo. Transparentar la información de la porción real de consumo en los productos individuales es un deber.(...) Nos interesa que nuestros consumidores estén y sean sanos. Tan convencidos estamos de ello, que desde hace varios años nuestras marcas dedican gran parte de su esfuerzo a promover la alimentación en porciones adecuadas, así como el equilibrio entre consumo y gasto calórico mediante actividad física. Por eso, con Costa, Ambrosoli, Carozzi y Vivo, hemos estado presentes en cientos de eventos deportivos, motivando a más de un millón de personas a sumarse al movimiento. |  | <https://www.carozzicorp.com/wp-content/uploads/2019/04/ECSA_Memoria_2018_Web.pdf> | 31-Dec-19 |
| A21 | Carozzi | Website industry | Discursive | Intended public health benefits | Policy does not work (from tobacco dystopia model) | Sin embargo, esta ley es incompleta, porque sólo regula los alimentos envasados, que equivalen apenas al 30% de la ingesta de los chilenos, dejando inexplicablemente fuera incluso a la comida rápida. Por otra parte, su Reglamento de Etiquetado frontal, basado en 100 grs y 100 ml, peca de sobre simplificación, ya que no entrega la información necesaria para que los consumidores puedan tomar buenas decisiones. Si bien el objetivo de esta Ley es loable y lo compartimos plenamente, se ha traducido en una política pública mal implementada. |  | <https://www.carozzicorp.com/wp-content/uploads/2019/04/ECSA_Memoria_2018_Web.pdf> | 31-Dec-19 |
| A22 | Carozzi | Website industry | Discursive | Frame the debate | Part of the solution | En Carozzi no estamos en contra de la Ley de Alimentos; al contrario, sentimos que tenemos el deber de colaborar en la solución de la problemática de la obesidad. Para esto hicimos una propuesta compartida con diferentes grupos de interés, que busca dar más y mejor información a los consumidores para que puedan tomar mejores decisiones. De acuerdo con la encuesta realizada por Cadem a nivel nacional, nuestra propuesta es respaldada por el 89% de las personas encuestadas. Por eso nos preguntamos, ¿No habrá llegado la hora de hacer cambios avanzando al Etiquetado 2.0? |  | <https://www.carozzicorp.com/wp-content/uploads/2019/04/ECSA_Memoria_2018_Web.pdf> | 31-Dec-19 |
| A23 | Carozzi | Website industry | Direct involvement and influence in policy | Lobby |  | jul 19, 2019 Fuente: El Mercurio Categoría: Gremiales Carozzi reactiva campaña para perfeccionar ley de etiquetado y entrega nueva propuesta A casi un mes de puesta en marcha de última fase más estricta de la normativa.  La firma de alimentos propuso al Gobierno un plan piloto para ofrecer a grupos de consumidores productos con 'discos pare' rediseñados y con información en base a porciones. También implementarán una aplicación de celular para escanear códigos. |  | <https://www.abchile.com/index.php?mact=News,cntnt01,detail,0&cntnt01articleid=681&cntnt01returnid=59&cntnt01returnid=59> | 31-Dec-19 |
| A24 | ChileAlimentos | Twitter | Discursive | Frame the debate | Part of the solution Personal responsibility | Chilealimentos @Chilealimentos Suivre Suivre @Chilealimentos Les presentamos nuestro programa Chile Crece Sano, donde se muestran las actividades que se han desarrollado e impulsa la Industria de los Alimentos en torno a Estilos de Vida Saludable. 11:29 - 17 juin 2019 |  | <https://twitter.com/Chilealimentos/status/1140687983982587911> | 27-Dec-19 |
| A25 | ChileAlimentos | Twitter | Information management | Amplification |  | Chilealimentos @Chilealimentos Suivre Suivre @Chilealimentos ESTUDIO SEÑALA QUE LAS FRAMBUESAS AYUDAN A CONTROLAR LA GLUCOSA https://goo.gl/hQh9hW  11:17 - 4 mars 2019 | Many other tweets like these | <https://twitter.com/Chilealimentos/status/1102649171805421568> | 27-Dec-19 |
| A26 | ChileAlimentos | Twitter | Information management | Amplification |  | Chilealimentos ‏@Chilealimentos Suivre Suivre @Chilealimentos INVESTIGADORES MEXICANOS DESCUBREN MOLÉCULA EN LA PALTA QUE AYUDARÍA A DISMINUIR ENFERMEDADES CRÓNICAS https://goo.gl/xMLza1  11:56 - 27 févr. 2019 | Many other tweets like these | <https://twitter.com/Chilealimentos/status/1100847234378330112> | 27-Dec-19 |
| A27 | ChileAlimentos | Website industry | Information management | Suppression |  | CINCO MITOS SOBRE LOS CONSERVANTES Y LOS MAL LLAMADOS ALIMENTOS “ULTRAPROCESADOS” (...) 3- “Entre más procesados sean los alimentos más daño causan a la salud”. Falso.  Realidad: procesar ciertos alimentos es fundamental para preservar y mantener seguro lo que consumimos. El mundo, y Colombia, se basa en marcos normativos que dictan entidades como el CODEX Alimentarius (organismo establecido por la Organización de las Naciones Unidas para la Alimentación y la Agricultura (FAO) y la OMS, y el Ministerio de Salud y Protección Social; quienes definen el actuar de la industria de alimentos. Según los expertos, la clasificación de alimentos “ultra procesados” es un error técnico, ya que no existe evidencia científica creíble que respalde este concepto.  De acuerdo con Camilo Montes, director de la Cámara de la Industria de Alimentos de la ANDI, “la definición de “ultraprocesados” sataniza a algunos grupos y, adicionalmente, ataca los esfuerzos de investigación y desarrollo en el país para la creación de nuevos alimentos. Un ejemplo de esto es la Bienestarina, un producto que ha traído grandes beneficios a miles de niños colombianos, pero que si nos basáramos en su nivel de procesamiento, este alimento formidable sería considerado un “ultraprocesado”. | They discuss about safety "Experts" is from a trade association | <http://www.chilealimentos.com/wordpress/cinco-mitos-sobre-los-conservantes-y-los-mal-llamados-alimentos-ultraprocesados/> | 27-Dec-19 |
| A28 | ChileAlimentos | Website industry | Discursive | Economy |  | Segunda fuerza exportadora de alimentos Chile es una Potencia Alimentaria y antecedentes hay de sobre. El sector es el segundo en generación de divisas y ha triplicado sus exportaciones en 15 años. |  | <https://chilealimentos.com/ventajas_categoria/segunda-fuerza-exportadora/> | 31-Dec-19 |
| A29 | Coca Cola | Media | Discursive | Frame the debate |  | Etiquetado y reforma tributaria Hay empresas de consumo que se han mostrado reticentes a la forma en cómo se ha implementado la norma de etiquetado nutricional en Chile, como Carozzi. Frente a esto, Vicuña reveló su posición. “Somos tremendamente respetuosos y cumplimos con las normas al pie del cayo. En el caso del etiquetado, cuando salió la ley nosotros nos anticipamos y cumplimos con todas las obligaciones. Esa es nuestra postura. Nos gusta que en todos nuestros productos esté la información que el consumidor necesita ver, como calorías, grasa o cantidad de azúcar. Nos parece bien informar a los consumidores”, dijo.  El presidente de la compañía también se refirió a la reforma tributaria y destacó que “lo único que queremos es que salga luego, porque mientras tanto se genera incertidumbre y eso no le hace bien al país y a los empresarios el no tener las reglas claras”. |  | <https://www.latercera.com/pulso/noticia/presidente-socio-coca-cola-embonor-consumidor-hoy-dia-ya-no-quiere-azucar/619705/> | 22-Nov-19 |
| A30 | Coca Cola | Media | Discursive | Frame the debate | self-regulation | En marzo, AB Chile redefinirá su postura respecto a la Ley de Etiquetado. Ese mes, el presidente del gremio, Rodrigo Álvarez, le propondrá al directorio un nuevo “rayado de cancha” respecto a la normativa. Es que las diferencias entre dos de sus socios han derivado en la necesidad de coordinar otro discurso común. Las divergencias entre Coca-Cola y Carozzi han permeado a la institución. El choque entre ambos actores -donde, en líneas generales, uno respalda la normativa, y el otro quiere profundas modificaciones- ha hecho que incluso existan peticiones de sanción sobre uno de ellos, mientras el otro evalúa permanentemente dejar el gremio. En el intertanto, ya se alista una reforma a los estatutos y un código de ética que los norme. (...) Todo, para lograr aunar posiciones en una soterrada disputa que ya suma varios años y capítulos, con -por un lado- un Carozzi desarrollando un intenso lobby para modificar la ley en pos -dicen- de combatir realmente la obesidad; y -por otro- un Coca-Cola valorando la normativa, y avanzando -como han repetido- a pasos agigantados en reducir los niveles de azúcar, y por consiguiente, en eliminar los sellos de sus productos. “Ambas empresas han estado en algún momento peleadas con todas las demás. |  | <https://www.latercera.com/pulso/noticia/la-disputa-carozzi-coca-cola-impulsa-una-nueva-definicion-gremial-frente-etiquetado/522607/> | 22-Nov-19 |
| A31 | Coca Cola | Media | Coalition management Discursive | Community Frame the debate | Part of the solution | "Un Mundo sin Residuos" fue lanzado en una interactiva actividad en la plaza de Renca. Ahí se anunció que Coca-Cola, Coca-Cola Andina y la Municipalidad de Renca, entregarán 17 contenedores de recolección de botellas plásticas que serán distribuidos en distintos puntos de la comuna, para dar acceso a aquellos vecinos que no tienen una red cercana de puntos limpios y contribuir así a aumentar las tasas de reciclaje. Esta iniciativa se suma a los dos años de trabajo en torno a educación ambiental y reciclaje, que se han desarrollado con niños bajo el Programa de Educación Ambiental. Esta labor se ha realizado en conjunto con la Empresa B Kyklos en 20 establecimientos de la comuna, para sensibilizar y educar a escolares sobre el reciclaje domiciliario, puntos de acopio y economía circular. (...) Por su parte, el alcalde de Renca, Claudio Castro, explicó que, "en Renca hemos avanzado decididamente en implementar medidas de reciclaje: hace dos años no teníamos iniciativas relacionadas y hoy ya contamos con una red de reciclaje comunal que comenzó gracias al apoyo de Coca-Cola Andina, con el trabajo de educación ambiental en nuestras escuelas públicas y la instalación de 14 puntos limpios en esos establecimientos" |  | <http://publirreportajes.latercera.com/quepasa/un-mundo-sin-residuos-la-iniciativa-de-coca-cola-para-fomentar-el-reciclaje/> | 22-Nov-19 |
| A32 | Coca Cola | Government | Direct involvement and influence in policy Discursive | Lobby Frame the debate | Part of the solution | Audiencia Datos de Audiencia Organismo público que informa: Camara De Diputados  Duración: 20 minuto(s)  Lugar: Valparaíso  Fecha Audiencia: 7 de mayo de 2019 10:30 Observaciones: Propuestas e iniciativas de Coca-Cola en materia de sustentabilidad (empaques y reciclabilidad).  Nombre: Sebastián Álvarez Ramírez Cargo: Diputado  Lobbistas o Gestores de Interés  Nombre: Fernanda Ramos  Calidad: Gestor Nacionalidad: Chile Trabaja para: S/I  Persona o Entidad a la que Representa: Coca-Cola de Chile S.A.   Nombre: Paola Calorio  Calidad: Gestor Nacionalidad: Chile Trabaja para: S/I  Persona o Entidad a la que Representa: Coca-Cola de Chile S.A. |  | https://www.infolobby.cl/Ficha/Audiencia/nr006ar125331 | 26-Dec-19 |
| A33 | Coca Cola | Government | Direct involvement and influence in policy Discursive | Lobby Frame the debate | Part of the solution | Audiencia Datos de Audiencia Organismo público que informa: Senado  Duración: 30 minuto(s)  Lugar: Santiago  Fecha Audiencia: 23 de septiembre de 2019 16:00 Observaciones: Con respecto al proyecto de ley que prohíbe el uso de plásticos pet en envases de menos de 250 cc, Coca-Cola Company da a conocer el esfuerzo que han realizado como empresa en la inversión de tecnología e innovación para el uso de los plásticos pet que ellos ponen en el mercado, para ir preparándose para la ley rep que entra en vigencia el 2022.  Nombre: Carmen Gloria Aravena Acuña Cargo: Senador  Lobbistas o Gestores de Interés  Nombre: Información En Proceso De Recepción Conforme A Convenio  Calidad: Gestor Persona o Entidad a la que Representa: Coca-Cola De Chile S.A. |  | <https://www.infolobby.cl/Ficha/Audiencia/nr00551921> | 26-Dec-19 |
| A34 | Coca Cola | Government | Direct involvement and influence in policy | Lobby |  | Audiencia Datos de Audiencia Organismo público que informa: Subsecretaría Del Medio Ambiente  Duración: 60 minuto(s)  Lugar: Santiago  Fecha Audiencia: 28 de febrero de 2019 15:00 Observaciones: Resumen de Acta: La reunión comenzó a las 15:00 Hrs. se verificaron los CI de los asistentes que participaron en la reunión. Conversaron de las iniciativas regulatorias para plásticos de un solo uso. En la reunión acompañaron a la Sra. Ministra las siguientes personas: Guillermo González, Jefe de la oficina de Implementación Legislativa y Economía Circular, Ximena Gonzalez, Daniel Vargas, Profesionales del la Oficina de Implementación Legislativa y Economía Circular, Sr. Gian Franco Raglianti, Abogado de la División Jurídica y el Sr. Guillermo Mac Person, Profesional de la Oficina de Comunicación y Prensa.  Nombre: María Carolina Schmidt Zaldivar Cargo: Ministro  Lobbistas o Gestores de Interés  Nombre: Miguel Flores Vargas  Calidad: Lobista Nacionalidad: Chile Trabaja para: Azerta Comunicaciones  Persona o Entidad a la que Representa: Coca-Cola de Chile S.A.   Nombre: Paola Calorio  Calidad: Gestor Nacionalidad: Chile Trabaja para: Coca-Cola de Chile S.A.  Persona o Entidad a la que Representa: Coca-Cola de Chile S.A.   Nombre: José Jaramillo  Calidad: Gestor Nacionalidad: Chile Trabaja para: Coca-Cola de Chile S.A.  Persona o Entidad a la que Representa: Coca Cola Chile S.A   Nombre: Daniel Vercelli  Calidad: Gestor Nacionalidad: Chile Trabaja para: Coca-Cola de Chile S.A.  Persona o Entidad a la que Representa: Coca Cola Chile S.A. |  | <https://www.infolobby.cl/Ficha/Audiencia/aw0023274481> | 26-Dec-19 |
| A35 | Coca Cola | Government | Direct involvement and influence in policy Discursive | Lobby Frame the debate | Part of the solution | Audiencia Datos de Audiencia Organismo público que informa: Camara De Diputados  Duración: 60 minuto(s)  Lugar: Valparaíso  Fecha Audiencia: 7 de mayo de 2019 11:30 Observaciones: Propuestas e iniciativas de Coca-Cola en materia de sustentabilidad (empaques y reciclabilidad)  Datos Sujeto Pasivo  Nombre: Nicolás Noman Garrido Cargo: Diputado  Lobbistas o Gestores de Interés  Nombre: Fernanda Ramos  Calidad: Gestor Nacionalidad: Chile Trabaja para: S/I  Persona o Entidad a la que Representa: Coca-Cola de Chile S.A.   Nombre: Paola Calorio  Calidad: Gestor Nacionalidad: Chile Trabaja para: S/I  Persona o Entidad a la que Representa: Coca-Cola de Chile S.A. |  | <https://www.infolobby.cl/Ficha/Audiencia/nr006ar125271> | 26-Dec-19 |
| A36 | Coca Cola | Government | Direct involvement and influence in policy Discursive | Lobby Frame the debate | Part of the solution | Audiencia Datos de Audiencia Organismo público que informa: Camara De Diputados  Duración: 30 minuto(s)  Lugar: Valparaíso  Fecha Audiencia: 7 de mayo de 2019 11:00 Observaciones: Propuestas e iniciativas de Coca-Cola en materia de sustentabilidad (empaques y reciclabilidad).  Datos Sujeto Pasivo  Nombre: Karin Luck Urban Cargo: Diputado  Lobbistas o Gestores de Interés  Nombre: Fernanda Ramos  Calidad: Gestor Nacionalidad: Chile Trabaja para: S/I  Persona o Entidad a la que Representa: Coca-Cola de Chile S.A.   Nombre: Paola Calorio  Calidad: Gestor Nacionalidad: Chile Trabaja para: S/I  Persona o Entidad a la que Representa: Coca-Cola de Chile S.A. |  | <https://www.infolobby.cl/Ficha/Audiencia/nr006ar124651> | 26-Dec-19 |
| A37 | Coca Cola | Twitter | Discursive | Frame the debate | Part of the solution | Coca-Cola Chile‏ Compte certifié @CocaColaCoCL #MedioAmbiente \| Conoce los proyectos que se desarrollan en Coca-Cola para ir en ayuda del agua. https://CokeURL.com/CompromisoAguaCL … 06:45 - 13 déc. 2019 | Many more Tweets - this account is all about Coke and its initatives in sustainability, but not all collected here as focus is on diet and nutrition | <https://twitter.com/CocaColaCoCL/status/1205499054748295169> | 27-Dec-19 |
| A38 | Coca Cola | Twitter | Discursive | Frame the debate | Part of the solution | Coca-Cola Chile Compte certifié  @CocaColaCoCL Suivre Suivre @CocaColaCoCL  #MedioAmbiente \| ¡Todo listo para el #6D, la mayor jornada de reforestación en Chile! Inscríbete aquí: https://CokeURL.com/TwListosPara6DCL … |  | <https://twitter.com/CocaColaCoCL/status/1202636372240216065> | 27-Dec-19 |
| A39 | Coca Cola | Twitter | Discursive | Frame the debate | Part of the solution | Coca-Cola Chile Compte certifié @CocaColaCoCL Suivre Suivre @CocaColaCoCL #MedioAmbiente \| Los desafíos y responsabilidades de las empresas en la lucha contra el cambio climático. https://CokeURL.com/TwAccionEmpresasCL … 05:05 - 29 nov. 2019 |  | <https://twitter.com/CocaColaCoCL/status/1200400402019168257> | 27-Dec-19 |
| A40 | Coca Cola | Twitter | Discursive | Frame the debate | Part of the solution | Coca-Cola Chile Compte certifié @CocaColaCoCL Suivre Suivre @CocaColaCoCL En este #DíaNacionaldelMedioAmbiente conoce el programa que está enseñando a miles de estudiantes sobre el reciclaje. https://CokeURL.com/TwSemillaCambioCL … 07:31 - 2 oct. 2019 |  | <https://twitter.com/CocaColaCoCL/status/1179403549689761798> | 27-Dec-19 |
| A41 | Coca Cola | Twitter | Direct involvement and influence in policy Discursive | Lobby Frame the debate | Part of the solution (gender equality) | Coca-Cola Chile Compte certifié @CocaColaCoCL Suivre Suivre @CocaColaCoCL #Comunidad \| ¡Es momento de empoderar a las mujeres! Esto se habló en una reunión entre empresarias, científicas, políticas y académicas. https://CokeURL.com/TwComunidadMujerCL … 05:58 - 11 sept. 2019 |  | <https://twitter.com/CocaColaCoCL/status/1171769992892825601> | 27-Dec-19 |
| A42 | Coca Cola | Twitter | Discursive | Frame the debate | Part of the solution Personal responsibility | Coca-Cola Chile Compte certifié  @CocaColaCoCL Suivre Suivre @CocaColaCoCL #MedioAmbiente \| Las personas, la sociedad y las empresas: todos debemos trabajar por un mundo sin residuos. https://CokeURL.com/MundoSinResiduosCL … 07:37 - 22 juil. 2019 |  | <https://twitter.com/CocaColaCoCL/status/1153313087233122305> | 27-Dec-19 |
| A43 | Coca Cola | Twitter | Information management Discursive | Amplification Frame the debate | Part of the solution | Coca-Cola Chile Compte certifié @CocaColaCoCL Suivre Suivre @CocaColaCoCL #Comunidad \| Educación y entretención: así se vive una visita a la planta de Coca-Cola en Concón. https://CokeURL.com/VisitaPlantaConConCL … 0:38 06:40 - 3 juil. 2019 | See the video - branding - Coca-Cola presented as a company that helps the environment, while the visit includes seeing the production line! | <https://twitter.com/CocaColaCoCL/status/1146413361367834624> | 27-Dec-19 |
| A44 | Coca Cola | Twitter | Discursive | Frame the debate | Part of the solution Physical activity | Coca-Cola Chile‏ Compte certifié @CocaColaCoCL Suivre Suivre @CocaColaCoCL Deporte, reciclaje y entretención en familia. Así se vivió la corrida de #ActitudRe. https://CokeURL.com/CorridaFamiliarCL … 0:30 09:44 - 21 janv. 2019 | See video - branding, distributing water and people have to recycle | <https://twitter.com/CocaColaCoCL/status/1087405494690631680> | 27-Dec-19 |
| A45 | Coca Cola | Twitter | Discursive | Frame the debate | Part of the solution | @CocaColaCoCL Suivre Suivre @CocaColaCoCL #Bienestar \| La buena noticia de Coca-Cola: el 95% de sus productos son bajos o sin calorías. http://CokeURL.com/DiferenciaPositivaCL … 0:31 11:47 - 14 janv. 2019 |  | <https://twitter.com/CocaColaCoCL/status/1084899771360468995> | 27-Dec-19 |
| A46 | Coca Cola | Website industry | Direct involvement and influence in policy Discursive | Lobby Frame the debate | Part of the solution (gender equality) | Coca-Cola y Comunidad Mujer: juntas por la transformación (…) La presencia de las diputadas Karin Luck y María José Hoffman y de la subsecretaria del nuevo Ministerio de Ciencias, Carolina Torrealba, le sumó otro matiz al debate: el del rol del estado y de las políticas públicas.. |  | <https://www.cocacoladechile.cl/historias/comunidad-coca-cola-y-comunidad-mujer-juntas-por-la-transformaci-n?utm_source=social&utm_medium=Twitter&utm_campaign=ccj-local-semana-2&utm_term=Reunion&utm_content=Mujeres> | 27-Dec-19 |
| A47 | Coca Cola | Website industry | Information management Discursive | Amplification Frame the debate | Part of the solution | Los niños y niñas de la Escuela República de Paraguay de Valparaíso vivieron una mañana inolvidable en la planta de Coca-Cola Embonor en Concón. Allí aprendieron sobre el proceso productivo de las bebidas y la importancia del reciclaje en el cuidado del planeta. | See the video - branding - Coca-Cola presented as a company that helps the environment, while the visit includes seeing the production line! | <https://www.cocacoladechile.cl/historias/comunidad-una-divertida-y-educativa-manana-en-la-planta-de-concon> | 27-Dec-19 |
| A48 | Coca Cola | Website industry | Discursive | Frame the debate | Part of the solution Physical activity | “Renca juega y aprende en comunidad” cierra un exitoso primer año Por: Journey Chile \| 1/21/2019 Confirmado: en 2019 seguirá el trabajo de la Fundación Fútbol Más en las escuelas de Renca. Así lo ratificaron la misma ONG, la municipalidad y Coca-Cola Andina durante la actividad que dio cierre a un exitoso 2018, en que cerca de 400 niñas y niños de 14 comunidades escolares aprendieron valores gracias al trabajo en la cancha. “Para Coca-Cola Andina es un orgullo participar en esta iniciativa, porque aporta directamente al bienestar emocional de nuestros vecinos en Renca, desarrollando valores y empatía en los niños a través del deporte. Este programa es fruto de una alianza público-privada, y es un ejemplo de las cosas positivas que se pueden hacer cuando se trabaja en forma colaborativa”, destacó Alejandro. |  | <https://www.cocacoladechile.cl/historias/comunidad-_renca-juega-y-aprende-en-comunidad-cierra-un-exitoso-primer-ano> | 27-Dec-19 |
| A49 | Coca Cola | Website industry | Discursive | Frame the debate | Part of the solution Physical activity | Alumnos y apoderados de la Escuela Alejandro Gorostiaga de Renca llegaron vestidos con tenidas deportivas para participar de una corrida familiar. En el marco de la campaña Actitud RE, la comunidad escolar compartió una jornada saludable y recicló todos los envases de agua con que se hidrataron los corredores. | See video - branding, distributing water and people have to recycle | <https://www.cocacoladechile.cl/historias/medio-ambiente-una-corrida-familiar-con-actitud-re> | 27-Dec-19 |
| A50 | Coca Cola | Twitter | Coalition management | Community |  | Desafío L. Chile @DesafioChile Suivre Suivre @DesafioChile Gracias @CocaCola_CL por apoyar con dos camiones con Kits de agua para ayudar a las personas damnificadas en Calama. #LevantemoselNorte 07:22 - 9 févr. 2019 |  | <https://twitter.com/DesafioChile/status/1094255100011446280> | 27-Dec-19 |
| A51 | Coca Cola | Website industry | Information management | Amplification | sweeteners | Samuel Durán, Nutricionista: "Que las bebidas hayan transitado al edulcorante es una excelente adaptación" Por: Journey Chile Chile ocupa el lugar 23 del mercado de los edulcorantes en el mundo y el gusto por lo dulce es un sello del país. Así lo expone el nutricionista e investigador Samuel Durán, quien explica por qué somos un país tan bueno para lo dulce y cuál es la tendencia de los endulzantes no calóricos. (...) Yo estoy de acuerdo con volver a lo natural, pero a las cantidades que comían nuestros abuelos, no a las cantidades “naturales” de hoy. Si hoy sacáramos los endulzantes, la gente no volvería a tomar agua y sólo subiría la ingesta de azúcar. En los ´80 la gente consumía azúcar en cantidades más bajas, menos del 5% de las calorías totales y hoy estamos cercanos al 10%. | Much information under 'bienestar' for sweeteners | <https://www.cocacoladechile.cl/historias/bienestar-samuel-dur-n-nutricionista-que-las-bebidas-hayan-transitado-al-edulcorante-es-una-excelente-adaptaci-n> | 27-Dec-19 |
| A52 | Coca Cola | Website industry | Information management | Amplification | sweeteners | La primera Jornada de Edulcorantes realizada en Chile fue una muestra más de la importancia que están adquiriendo los endulzantes no calóricos, ad portasde cumplirse tres años de la promulgación de la Ley de Etiquetado, normativa que incentivó la reformulación en recetas de alimentos y bebidas. (...) Tras promulgarse en 2016 la Ley de Etiquetado de Alimentos, la industria alimentaria del país comenzó a adecuarse a los nuevos parámetros exigidos por la autoridad. Muchos productos que antes contenían azúcar, por ejemplo, fueron reformulados con edulcorantes no calóricos. Surgió así un renovado interés por desarrollar investigaciones sobre endulzantes distintos al azúcar y en ese marco se celebró en Santiago la Primera Jornada de Edulcorantes, una charla para profesionales convocada por la **Carrera de Nutrición y Dietética de la Universidad Santo Tomás.** (...) “Chile está en el lugar 23 del mercado mundial de edulcorantes y eso es harto para un país pequeño como el nuestro. Pero todavía seguimos siendo de los mayores consumidores de azúcar del mundo, con 142,7 gramos per cápita al día, según la consultora Euromonitor”, explica Samuel Durán, vicepresidente del Colegio de Nutricionistas de Chile. Gran parte de la explicación, según Samuel, es porque Chile es un país más dulce que el promedio ya que “venimos de una generación donde nuestras mamaderas y papillas estaban llenas de azúcar, nuestros jugos de frutas eran con azúcar, entonces después de 25 años o más, tenemos apetencia por los sabores dulces, porque nos formaron de esa manera”. |  | <https://www.cocacoladechile.cl/historias/bienestar-hablemos-sobre-endulzantes> | 30-Dec-19 |
| A53 | Coca Cola | Website industry | Information management Discursive | Amplification Frame the debate | sweeteners  part of the solution personal responsiblity | El exitoso primer año de Coca-Cola Sin Azúcar Por: Journey Chile \| 2/4/2019 Coca-Cola Sin Azúcar es tan parecida a Coca-Cola Sabor Original, que sólo la robot especializada M.A.R.T.A las puede diferenciar. A un año de su lanzamiento, la bebida se ha instalado en más de la mitad de los hogares chilenos que consumen gaseosas. Estas son las claves de su éxito. (...) La refrescante Coca-Cola Sin Azúcar está celebrando su primer cumpleaños este verano (...) sus ventas crecieron un 20% en estos doce primeros meses, llegando a 374 mil nuevos hogares. En otras palabras, se posicionó en más de la mitad de las casas de los chilenos que compran gaseosas. “Los consumidores chilenos están demandando cada vez más productos sin azúcar y Coca-Cola Sin Azúcar se instaló como una propuesta atractiva, con un nombre más claro y un sabor igual de rico y refrescante”, explica Paula Catanzaro, Sparkling Senior Brand Manager de Coca-Cola South Latin. (...) Chile es el noveno país del mundo, y el primero de Latinoamérica, donde Coca-Cola tiene disponible el mayor mix de bebidas bajas y sin calorías, en línea con consumidores que prefieren hidratarse de forma cada vez más saludable. Paula Catanzaro destaca que actualmente el 98% de los productos del portafolio de Coca-Cola no tiene sellos y la idea es seguir en el camino de perfeccionar sus recetas, porque “este trabajo es como la tecnología, que está constantemente mejorando las cosas, y nosotros queremos seguir mejorando el sabor y composición de nuestras bebidas, porque es lo que piden los consumidores”. | See also other webpages under 'bienestar' - most information on sweeteners: https://www.cocacoladechile.cl/historias/bienestar-bebidas-sin-az-car-alternativas-para-todos-los-paladares https://www.cocacoladechile.cl/historias/bienestar-Acesulfame-K,-siempre-bien-acompanado https://www.cocacoladechile.cl/historias/bienestar-competencia-sabor-similitudes-diferencias https://www.cocacoladechile.cl/historias/bienestar-Con-K-de-potasio  https://www.cocacoladechile.cl/historias/bienestar-aspartame-nota-seguridad https://www.cocacoladechile.cl/historias/bienestar-aspartame-sabor-intacto https://www.cocacoladechile.cl/historias/bienestar-tres-conceptos-seguridad-aspartame https://www.cocacoladechile.cl/historias/bienestar-revolucion-endulzantes https://www.cocacoladechile.cl/historias/bienestar-innovacion-bajas-calorias https://www.cocacoladechile.cl/historias/bienestar-nueva-formula-para-el-exito | <https://www.cocacoladechile.cl/historias/bienestar-el-exitoso-primer-ano-de-coca-cola-sin-azucar> | 30-Dec-19 |
| A54 | Coca Cola | Website industry | Information management | Amplification Suppression | sweeteners | Edulcorantes: con respaldo científico internacional Científicos de todo el mundo se dieron cita en Lisboa en la “Jornada Internacional sobre Edulcorantes bajos en o sin calorías”. Lisboa fue el escenario para que representantes de sociedades y fundaciones de nutrición y dietética de Europa y Latinoamérica de reunieran, en el marco de la “Jornada Internacional sobre Edulcorantes bajos en o sin calorías”. (...) Además, destacaron los resultados de un estudio realizado por Samuel Durán, vicepresidente de la Sociedad Chilena de Nutrición, que descarta la relación entre el consumo de estos ingredientes y el aumento de peso. Realizado sobre 1224 jóvenes universitarios de cuatro países, este trabajo no encontró ninguna asociación entre el uso de edulcorantes no nutritivos o no calóricos y el riesgo de sobrepeso u obesidad y que, en ese sentido, su uso está en sintonía con las políticas públicas de todos los países que promueven reemplazar el azúcar para disminuir la ingesta calórica. (...) Otro de los aspectos abordados fue la ausencia de evidencia científica que relacione el consumo de edulcorantes con el aumento de apetito. Por el contrario, a juicio de los expertos los endulzantes sin calorías contribuyen a alcanzar la apetencia de algo dulce y a disminuir la ingesta de calorías cuando se usan para reemplazar azúcares. Y en esa misma línea, Samuel Durán fue tajante al desestimar también el mito de que estas sustancias provocarían cambios a nivel intestinal. |  | <https://www.cocacoladechile.cl/historias/bienestar-edulcorantes-respaldo-cientifico> | 30-Dec-19 |
| A55 | Coca Cola | Website industry | Information management | Amplification | sweeteners | los edulcorantes pueden ser consumidos por embarazadas, niños o adultos”, aclara Susana Socolovsky, Consultora en Asuntos Científicos y Regulatorios, que estuvo en Chile para participar en el simposio “Reducción del Contenido de Azúcar en Alimentos: Desde la Evidencia a la Acción”, organizado por la Sociedad Chilena de Ciencia y Tecnología de los Alimentos y la Sociedad Chilena de Nutrición. | Same person who spoke about FOPL in Colombia | <https://www.cocacoladechile.cl/historias/bienestar-hay-que-saber-de-edulcorantes> | 30-Dec-19 |
| A56 | Coca Cola | Website industry | Coalition management Discursive | Community Frame the debate | Part of the solution | Dos días de trekking para reforestar y contribuir al agua Por: Journey Chile Un grupo de jóvenes universitarios se puso la camiseta por el medioambiente y participó durante dos días en un trekking de sensibilización ambiental y reforestación en una reserva natural del cajón del Maipo, vital para el cuidado del agua de la que se abastecen los santiaguinos.  La tarea era ardua, pero la vocación medioambiental fue más grande porque la meta valía la pena: reforestar y proteger bosques nativos, humedales altoandinos y zonas ribereñas de la cuenca del río Maipo. La convocatoriam lanzada por Coca-Cola Chile y la ONG The Nature Conservancy con apoyo de la Corporación Cultiva -que ejecuta proyectos y programas de reforestación y educación ambiental- reunió a un grupo de 70 jóvenes voluntarios que, en dos días, recorrió los cerros y plantó árboles en sectores de la Reserva Nacional Río Clarillo, una zona prioritaria para el abastecimiento del agua que se consume en Santiago (...) La apuesta del proyecto de The Nature Conservancy y Coca-Cola Chile es grande, porque -en pleno año de megasequía- apunta a restaurar, reforestar y proteger distintos hábitats naturales en la parte alta de la cuenca del río Maipo. |  | <https://www.cocacoladechile.cl/historias/medio-ambiente-dos-dias-de-trekking-para-reforestar-y-contribuir-al-agua> | 30-Dec-19 |
| A57 | Coca Cola | Website industry | Coalition management Discursive | Community Frame the debate | Part of the solution | Semilla del cambio: Alumnos de la región de Valparaíso aprenden a reciclar Por: Journey Chile Colegios de Valparaíso y Viña del Mar se sumaron al programa de reciclaje y cuidado del medioambiente que llevan adelante Coca-Cola Embonor y Kyklos. La alianza genera conciencia en la comunidad sobre la importancia de proteger el planeta con la mirada puesta en el futuro. | See picture: branding | <https://www.cocacoladechile.cl/historias/medio-ambiente-semilla-del-cambio-alumnos-de-la-regi-n-de-valpara-so-aprenden-a-reciclar> | 30-Dec-19 |
| A58 | Coca Cola | Website industry | Discursive | Economy Frame the debate | Part of the History Part of the solution | Desde entonces, hace ya 75 años, Coca-Cola forma parte de la vida de los chilenos. Hoy cuenta con un amplio portafolio de más de 60 productos para satisfacer los gustos y necesidades de los consumidores. La Compañía ha estado presente en las mesas de muchas generaciones, refrescando a cada uno de los consumidores, siempre comprometida con el desarrollo social y económico del país.(...) Hoy, con una inversión anual de US$ 250 millones, el aporte económico anual del grupo en el país es de US$2.400 millones, lo que equivale a casi el 1% del PIB nacional. Asimismo, a través de toda la cadena de valor, genera 103 mil empleos directos e indirectos, cifra que corresponde al 1,3% de la fuerza laboral total (...) Éste fue el inicio de la producción de bebidas con cero o bajas calorías, que comenzaron a producirse bajo la marca Coca-Cola Diet, hoy Coca-Cola Light. A inicios de la década de 2000, Coca-Cola fue aún más allá: en 2006 lanzó Coca-Cola Zero, y en 2013, la versión Life. | See also here: https://www.cocacoladechile.cl/nuestra-compania/el-sistema-coca-cola | <https://www.cocacoladechile.cl/historias/history-coca-cola-en-chile-75-anos-haciendo-una-diferencia-positiva> | 30-Dec-19 |
| A59 | Coca Cola | Website industry | Discursive | Frame the debate | Ministry of Health | Planta Coca-Cola Talca, un entorno con sello saludable 5/6/2019 Todos queremos trabajar en un lugar donde te cuidan. La Planta Talca de Coca-Cola Embonor se propuso velar por la salud mental y física de sus asociados. Y el objetivo se logró con tan buenos resultados, que le significó recibir una importante acreditación de la autoridad sanitaria. La Planta de Talca de Coca-Cola Embonor se ha convertido en todo un ejemplo para el Sistema Coca-Cola, por propiciar un entorno feliz y saludable para sus trabajadores. Tanto así, que la Secretaría Regional Ministerial de Salud del Maule decidió distinguir ese trabajo y acreditó por dos años a la planta como un “Lugar de trabajo promotor de salud”. |  | <https://www.cocacoladechile.cl/historias/nuestra-gente-planta-coca-cola-talca--un-entorno-con-sello-saludable> | 30-Dec-19 |
| A60 | Coca Cola | Website industry | Discursive | Frame the debate | Part of the solution | gestionamos el negocio con una visión de futuro amplia, tomando decisiones que permitan no solo generar la mayor rentabilidad para nuestros accionistas, sino también brindar bienestar en las comunidades de las que formamos parte, influir positivamente en el cuidado del medioambiente, desarrollando comercio justo con nuestros clientes y proveedores, y fortaleciendo un ambiente de trabajo de bienestar para nuestros colaboradores. |  | <http://www.koandina.com/pagina.php?p=mensaje-presidente> | 31-Dec-19 |
| A61 | Coca Cola | Website industry | Discursive | Frame the debate | Part of the solution Self-regulation | Etiquetado con información nutricional Chile Chile puso en vigencia la Ley Nº 20.606 de Etiquetado, que obliga a rotular los envases con el sello de advertencia “ALTO EN”, que indica que ese producto contiene altos niveles de azúcares, sodio, grasas saturadas y/o calorías; restringe la publicidad dirigida a menores de 14 años de esos alimentos, además de su venta, promoción y entrega gratuita en establecimientos educativos. La normativa fue pionera en el mundo por su nivel de exigencias. En Coca-Cola Andina interpretamos la ley como una oportunidad para desarrollar un portafolio con amplitud de versiones de bebidas regulares bajas en azúcar y sin azúcar. Fuimos la primera Compañía de bebidas a nivel mundial que se comprometió a colocar el contenido de calorías y cantidad de azúcares, grasas totales y sodio en las etiquetas de los productos de nuestro portafolio. Para Coca-Cola Andina es clave tener un rol activo y responsable con nuestra comunidad y por esta razón proporcionamos información nutricional objetiva, significativa y comprensible sobre todos nuestros productos. Sabemos de la importancia de informar a los consumidores a través de nuestras etiquetas. El etiquetado nutricional basado en la evidencia permite a nuestros consumidores tomar decisiones que satisfagan sus requerimientos nutricionales y de energía individuales como de sus familias.  Marketing responsable Contamos con una política de marketing responsable, que estipula que no se dirige publicidad a niños de menos de 12 años para ninguno de los productos de la Compañía, no se pauta publicidad en medios cuya audiencia de niños menores de 12 años es mayor a un 35% y no se muestra a niños menores de 12 años bebiendo ninguno de los productos sin la presencia de un adulto responsable. Utilizamos las Guías Diarias de Alimentación (GDA), que son una herramienta de información nutricional presentada en formato de tabletas en el etiquetado de los productos. De acuerdo con la política global The Coca-Cola Company todas las etiquetas (excepto vidrio y agua) deben contener las GDA. En América Latina, presentamos la cantidad de calorías, junto con el porcentaje del Valor Diario (%VD) en el frente de los empaques, siendo coherente con el compromiso de ofrecer a los consumidores información nutricional transparente en sus productos. Además, un panel de información nutricional provee datos adicionales sobre proteínas, carbohidratos, fibra y, cuando el producto los contiene, minerales y vitaminas. (...) Los endulzantes no calóricos utilizados en las bebidas gaseosas light/cero de la Compañía son seguros para toda la población, incluyendo niños mayores de dos años, mujeres embarazadas y en período de lactancia. Esto está avalado por organismos internacionales, tales como la Administración Federal de Drogas y Alimentos de EEUU (FDA, por sus siglas en inglés), la Organización de las Naciones Unidas para la Alimentación y la Agricultura (FAO), la Organización Mundial de la Salud (WHO), el Comité Mixto de Expertos en Aditivos Alimentarios de la FAO/WHO (JECFA) y la Autoridad Europea de Seguridad Alimentaria (EFSA). |  | <http://www.koandina.com/uploads/Memoria%20Anual%20Integrada%202018.pdf> | 31-Dec-19 |
| A62 | Coca Cola | Website industry | Coalition management | Community |  | INICIATIVAS Chile • Programa “Renca, Juega y aprende en comunidad”, en alianza con Fútbol Más y la Municipalidad de Renca • Educación ambiental en Colegios en alianza con Kyklos y la Municipalidad de Renca, Maipú, Puente Alto y San Joaquín • Interescolar Ambiental Colegios ACTITUD RE: concurso de reciclaje de botellas PET y tapas retornables en 100 colegios de la RM • Programa de Recuperación de espacios en barrios con Fútbol Más en Santa Emilia, Renca • Programa de puertas abiertas: Cine CocaCola Andina; Fábrica de la felicidad • Cumplimos tres años de alianza con la Red de alimentos • Certificación APL piloto REP con CENEM. De la agencia de cambio climático, el Ministerio de Medio Ambiente y el Ministerio de Energía |  | <http://www.koandina.com/uploads/Memoria%20Anual%20Integrada%202018.pdf> | 31-Dec-19 |
| A63 | Coca Cola | Website industry | Discursive | Frame the debate | Part of the solution Self-regulation | Siguiendo las preferencias de los consumidores, en conjunto con The Coca-Cola Company, la empresa ha efectuado grandes esfuerzos por reducir en forma significativa, los niveles de azúcar en su portafolio. Mediante el lanzamiento de nuevas formulaciones y a través de nuevas opciones de bebidas, ha reducido en forma significativa la cantidad de azúcar utilizada en los procesos de embotellado. A fines de 2017, las ventas de bebidas con ‘bajas en’ o ‘sin calorías’ en Chile, alcanzó a casi el 80% de los SKU ofrecidos. |  | <http://www.embonor.cl/wps/wcm/connect/embonor/Sitio/Home/Sustentabilidad/Bienestar/Amplio+Portafolio+de+Productos/?subs=Amplio%20Portafolio%20de%20Productos> | 31-Dec-19 |
| A64 | Coca Cola | Website industry | Coalition management | Community |  | ¡Disfruta el sabor de nuestra Big Mac y ayuda! Contribuir a un futuro con mayores oportunidades es uno de los objetivos de la novedosa campaña Corazón de Big Mac, impulsada este 2019 por McDonald’s en Chile. La iniciativa, que tiene como rostro a Sergio Lagos, busca recolectar fondos a través de la venta de cupones digitales de hamburguesas Big Mac, para aportar al desarrollo de los programas sociales de la Fundación Coanil y la Fundación para la Infancia Ronald McDonald. Para colaborar, los antiguos y nuevos usuarios de la app de McDonald’s pueden encontrar, entre el 2 y el 15 de diciembre, el cupón Corazón de Big Mac, con el que podrán entregar su apoyo a las fundaciones dentro de todo el país. (...) Por su parte, la Fundación para la Infancia Ronald McDonald actualmente mantiene seis salas familiares construidas en hospitales públicos y, próximamente, inaugurará una nueva instalación en el Hospital de Rancagua. (....) La Fundación para la Infancia Ronald McDonald está trabajando también sobre ruedas a través de su consultorio pediátrico móvil. El programa, lanzado en 2017, busca aportar a la atención médica en zonas rurales a través de la habilitación de un camión como hospital móvil. Este ya ha atendido a 4 mil niños de comunas de las regiones del Maule y O’Higgins. |  | <https://www.mcdonalds.cl/noticias/corazondebigmac> | 31-Dec-19 |
| A65 | Coca Cola | Other | Direct involvement and influence in policy | Actors |  | PROGRAMA CONJUNTO FAO/OMS SOBRE NORMAS ALIMENTARIAS COMISIÓN DEL CODEX ALIMENTARIUS 42.o período de sesiones Ginebra (Suiza) 8–12 de julio de 2019 INFORME DE LA 45.a REUNIÓN DEL COMITÉ DEL CODEX SOBRE ETIQUETADO DE LOS ALIMENTOS Ottawa (Ontario, Canadá) 13–17 de mayo de 2019 REP19/FL Appendix I 21 Appendix I LISTA DE PARTICIPANTES Mrs Marisol Figueroa Barrientos Gerente General y Gerente Técnico de Alimentos y Bebidas de Chile A.G. (AB Chile) Alimentos y Bebidas de Chile A.G. - AB Chile Los Militares 6191, oficina 71, piso 7, Las Condes. Santiago Chile Email: mfigueroa@abchile.com Mrs Gisela Rodríguez Rideau Gerente Asuntos Científicos y Regulatorios Coca-Cola Avenida Kennedy 5757, piso 12. Las Condes Santiago Chile Tel: +56 2 233834209 Email: gisrodriguez@coca-cola.com |  | <http://www.fao.org/fao-who-codexalimentarius/sh-proxy/en/?lnk=1&url=https%253A%252F%252Fworkspace.fao.org%252Fsites%252Fcodex%252FMeetings%252FCX-714-45%252FFinal%252520Report%252FREP19_FLs.pdf> | 01-Nov-19 |
| A66 | Coca Cola Nestle | Twitter | Coalition management Discursive | Community Frame the debate | Part of the solution (sustainibility) | ACCIÓN Empresas ‏@ACCION_Empresas Suivre Suivre @ACCION_Empresas Cuatro de nuestras empresas socias: @aguas_andinas @NestleCL @AngloAmericanCL y @CocaColaCoCL, son parte del primer Fondo del Agua de la Región Metropolitana, el cual busca resguardar la seguridad hídrica del río Maipo.  #escasezhídrica Lee más aquí: https://bit.ly/32nXTLA  11:28 - 16 oct. 2019 | Other tweets as well - but not about diet and nutrition, so not all data collected here | <https://twitter.com/ACCION_Empresas/status/1184536541315686400> | 27-Dec-19 |
| A67 | Grupo Bimbo | Media | Coalition management | Opposition fragmentation |  | Este viernes, Canal 13 e Ideal, parte de Grupo Bimbo, comunicaron su “completo acuerdo” en el contexto de la millonaria demanda que la empresa de alimentos mantenía contra la estación televisiva, tras la emisión, en 2013, de un reportaje que cuestionaba la calidad nutricional de algunos de sus productos. Mediante un comunicado conjunto, las compañías valoraron el acuerdo y los esfuerzos hechos para acercar posiciones, aunque declinaron referirse a los términos del convenio. Cabe recordar que la justicia acogió parcialmente la demanda de Ideal y condenó al canal a pagar más de $4.000 millones, monto que posteriormente fue rebajado a $1.000 millones y que la estación también apeló. |  | <https://www.latercera.com/pulso/noticia/ideal-canal-13-llegan-acuerdo-tras-millonaria-demanda-reportaje/838581/> | 22-Nov-19 |
| A68 | ILSI | Twitter | Information management | Amplification |  | Ilsi Sur-Andino @IlsiSurAndino Suivre Suivre @IlsiSurAndino Seminario Cambio Climático y su potencial impacto en la industria de la nutrición Organizan ILSI Sur-Andino y UNAB, 24 octubre, Gratuito previa inscripción contacto@ilsisurandino.cl 07:11 - 16 oct. 2019 |  | <https://twitter.com/IlsiSurAndino/status/1184471832973459468> | 27-Dec-19 |
| A69 | ILSI | Twitter | Information management | Amplification |  | Ilsi Sur-Andino ‏@IlsiSurAndino Suivre Suivre @IlsiSurAndino IV Jornada de Nutrición y Dietética: Nutrición y alimentación basada en la evidencia: implicancias en cáncer" - https://mailchi.mp/ilsisurandino/iv-jornada-de-nutricin-y-diettica-nutricin-y-alimentacin-basada-en-la-evidencia-implicancias-en-cncer … 11:33 - 23 août 2019 |  | <https://twitter.com/IlsiSurAndino/status/1164968933050527745> | 27-Dec-19 |
| A70 | ILSI | Twitter | Information management | Amplification |  | @IlsiSurAndino Suivre Suivre @IlsiSurAndino Curso "Avances en microbioma intestinal, nutrición y salud - https://mailchi.mp/ilsisurandino/curso-avances-en-microbioma-intestinal-nutricin-y-salud  11:06 - 10 juin 2019 |  | <https://twitter.com/IlsiSurAndino/status/1138145345928605703> | 27-Dec-19 |
| A71 | ILSI | Twitter | Information management | Amplification |  | Ilsi Sur-Andino ‏@IlsiSurAndino Suivre Suivre @IlsiSurAndino III Foro de Comunicación Responsable en Ciencia y Salud - https://mailchi.mp/ilsisurandino/iii-foro-de-comunicacin-responsable-en-ciencia-y-salud … 09:10 - 4 juin 2019 |  | <https://twitter.com/IlsiSurAndino/status/1135941963528757249> | 27-Dec-19 |
| A72 | ILSI | Twitter | Information management | Amplification |  | Ilsi Sur-Andino @IlsiSurAndino Suivre Suivre @IlsiSurAndino Jornada de Investigación en Nutrición y Alimentos - https://mailchi.mp/ilsisurandino/jornada-de-investigacin-en-nutricin-y-alimentos … 08:33 - 10 mai 2019 | See programme See also here: https://mailchi.mp/ilsisurandino/jornada-de-investigacin-en-nutricin-y-alimentos | <https://twitter.com/IlsiSurAndino/status/1126872953470242816> | 27-Dec-19 |
| A73 | ILSI | Twitter | Information management | Amplification |  | Ilsi Sur-Andino  @IlsiSurAndino Suivre Suivre @IlsiSurAndino #IntegridadCientífica o la importancia de hacer bien lo que hacemos. Dra. Andrea Leisewitz. Los eperamos viernes 22 de marzo a las 10 am en ILSI SurAndino (Pérez Valenzuela 1098 of 101 Providencia). Inscripciones contacto@ilsisurandino.cl 07:31 - 14 mars 2019 |  | <https://twitter.com/IlsiSurAndino/status/1106201169838436355> | 27-Dec-19 |
| A74 | ILSI | Website industry | Information management | Amplification |  | IV JORNADA NUTRICIÓN Y DIETÉTICA USEK • NUTRICIÓN Y ALIMENTACIÓN BASADA EN LA EVIDENCIA: IMPLICANCIAS EN CÁNCER Universidad SEK, Auditorio Felipe Segovia., Fernando Manterola #0789 Providencia, Santiago 31 de agosto de 2019 de 8:30 am a 2:00 pm |  | <https://welcu.com/cinthia-quezada/iv-jornada-nutricion-y-dietetica-usek-nutricion-y-alimentacion-basada-en-la-evidencia-implicancias-en-cancer?fbclid=IwAR2akk8XOjw2q4aWAxwkkpQglCoj-FD-LLsQ4oqv-m858OFGzaL9XHaSAkE> | 27-Dec-19 |
| A75 | ILSI | Website industry | Information management | Amplification |  | Curso: Avances en Microbioma intestinal, nutrición y salud Viernes 28 de junio Dr. Daniel Garrido  LUGAR Pérez Valenzuela 1098, Oficina 101, Providencia   HORARIO 10:00 a 12:30 horas   Valores Empresas $20.000 Profesionales independientes $6.000 Estudiantes $3.000 |  | <https://mailchi.mp/ilsisurandino/curso-avances-en-microbioma-intestinal-nutricin-y-salud> | 27-Dec-19 |
| A76 | ILSI | Facebook | Information management | Amplification |  | International Life Sciences Institute - ILSI 8 avril ·  ILSI South Andean signed an agreement with Universidad Santo Tomás in Santiago, Chile to host a series of lectures for the students of Nutrition and Dietetics. The first in the series will take place tomorrow April 9. @IlsiSurAndino |  | <https://www.facebook.com/301041540049017/posts/1274537496032745/> | 27-Dec-19 |
| A77 | ILSI | Website industry | Information management | Amplification |  | Jornadas Universitarias sobre Nutrición, Concepción UBICACIÓN: Auditorio UST Concepción Tema: “Relación entre condición física y salud en párvulos y escolares”. Expositor: Dra. Johana Soto Horario: 11:30am Dirección: Auditorio UST Concepción Av. Prat 879, Concepción-Chile.  Descripción del Curso:  Chile a nivel mundial presenta una de las prevalencias más altas de sobrepeso y obesidad infantil, con más del 50% de su población, desde el nivel parvulario. Por otra parte, se ha observado una deficiente condición física en niños mayores de 8 años y adolescentes. A partir de estos antecedentes, resulta crucial comprender y conocer si a nivel preescolar ya existe una relación entre condición física y marcadores de salud no invasivos.  Si quieres leer más sobre nuestras Jornadas Universitarias, haz click acá: http://www.ilsisurandino.cl/2019/03/19/jornadas-universitarias-sobre-nutricion |  | <http://www.ilsisurandino.cl/events/jornadas-universitarias-sobre-nutricion-concepcion/> | 27-Dec-19 |
| A78 | ILSI | Website industry | Information management | Amplification |  | JORNADAS UNIVERSITARIAS SOBRE NUTRICIÓN 19 Marzo, 2019 Jornadas universitarias Este año en ILSI Sur-Andino estamos incentivando a los estudiantes jóvenes del área de nutrición de manera de apoyar a futuros profesionales en temas que lideran en el ámbito de nutrición y salud. Actualmente, estamos organizando Charlas y Jornadas Universitarias en Universidad SEK y Universidad Santo Tomás, con expertos que son parte de ILSI y que nos apoyan en la realización de estas actividades.  Ya contamos con 2 fechas confirmadas: Martes 9 de abril (crear link al banner)  Lugar: Universidad Santo Tomás, Concepción. Horario: 11:30 horas Tema: “Relación entre condición física y salud en párvulos y escolares”. Expositor: Dra. Johana Soto Viernes 26 de abril (crear link al banner)  Lugar: Universidad Santo Tomás, Santiago Horario: 11:00 horas Tema: “Relación entre condición física y salud en párvulos y escolares”. Expositor: Dra. Johana Soto |  | <http://www.ilsisurandino.cl/2019/03/19/jornadas-universitarias-sobre-nutricion/> | 27-Dec-19 |
| A79 | ILSI | Website industry | Coalition management | Internal |  | Las industrias que participan en ILSI Sur-Andino actualmente son Unilever, Tres Montes Luchetti, Monsanto, Nestlé, Kraft, Danisco-Dupont, DSM, Coca-Cola |  | <http://www.ilsisurandino.cl/sitio/ilsi-sur-andino/sobre-ilsi> | 31-Dec-19 |
| A80 | McDonald's | Media | Coalition management Discursive | Governement Frame the debate |  | Ministra de Medio Ambiente entrega sello #ChaoBombillas a McDonald´s  La campaña fue lanzada en septiembre del año pasado por la cartera y apunta a un océano libre de plásticos. La Ministra del Medio Ambiente, Carolina Schmidt, visitó uno de los 84 restaurantes de McDonald´s, donde recorrió las instalaciones y se interiorizó en los procesos y políticas que la empresa ha puesto en práctica en materia sustentable, y entregó a la empresa el sello “ChaoBombillas” campaña que inició la cartera hace un año por un océano libre de plásticos. |  | <https://www.latercera.com/pulso/noticia/ministra-medio-ambiente-entrega-sello-chaobombillas-mcdonalds/813569/> | 22-Nov-19 |
| A81 | Nestle | Media | Coalition management Discursive | Community Frame the debate |  | Empresas firman este martes pacto con la Intendencia para proteger el río Maipo  Entre las compañías que se sumarán al Fondo del Agua destacan Nestlé, Aguas Andinas y la minera Anglo American. La creación del primer Fondo del Agua del país, que tiene por objeto generar proyectos de restauración para toda la cuenca del río Maipo, firmarán el martes un grupo de empresas con la Intendencia Metropolitana. Esto, con la finalidad de responder al complicado escenario de escasez hídrica que afecta a la región.  Este Fondo del Agua es liderado por The Nature Conservancy (TNC) en representación de la Alianza Latinoamericana de Fondos de Agua. Además, cuenta con la presencia del gobierno regional, la Asociación de Municipalidades rurales (AMUR), Aguas Andinas, Nestlé, la ONG Adapt Chile, Anglo American, Federación Nacional de Cooperativas de Servicios Sanitarios (Fesan) y la Confederación de Canalistas de Chile. |  | <https://www.latercera.com/pulso/noticia/empresas-firman-hoy-pacto-la-intendencia-proteger-rio-maipo/861178/> | 22-Nov-19 |
| A82 | Nestle | Profesionnal assoc | Information management | Amplification |  | SOCHINUT informa los ganadores del Concurso de Proyectos de Investigación 2019 15.11.2019  SOCHINUT - TETRA PAK Claudia Vega Soto "Intervención interdiciplinaria destinada a mejorar la capacidad y función muscular de mujeres adultas mayores diagnosticadas con sacopenia pertenecientes a un hogar de larga estadía de la comuna de Valparaíso"   SOCHINUT - NESTLÉ Eugenia Morselli "Saturated Fatty Acids Cause Lisosomal Damage in Hypothalamic Neurons"   SOCHINUT - NESTLÉ Sandra Lopez Arana "Caracterización de las dificultades alimentarias y estado nutricional en lactantes y preescolares atendidos en un centro del sistema de salud privado"   SOCHINUT - NESTLÉ Loreto Rojas Sobarzo "Efecto de la ingesta de sal de mesa no yodada en la yoduria de sujetos adultos sanos"  Felicitaciones. |  | <https://www.sochinut.cl/single-post/2019/11/15/SOCHINUT-informa-los-ganadores-del-Concurso-de-Proyectos-de-Investigaci%C3%B3n-2019> | 27-Nov-19 |
| A83 | Nestle | Profesionnal assoc | Information management | Amplification |  | Jornada de actualización en Nutrición Clínica September 11, 2019 Organiza: Clínica Alemana Temuco Auspiciadores: ...Nestlé... | Many other industry participants - see the doc | <https://www.colegiodenutricionistas.cl/single-post/2019/09/11/Jornada-de-actualizaci%C3%B3n-en-Nutrici%C3%B3n-Cl%C3%Adnica> | 29-Nov-19 |
| A84 | Nestle | Profesionnal assoc | Information management | Amplification |  | IV Curso de Nutricionistas al día de Clínica Las Condes Organiza: Clinica Las Condes, Departamento de Nutricion, Faculdad de Medicina, Universidad de Chile Patrocina: Colegio de Nutricionistas Universitarios de Chile A.G Auspicia: …. Nestle... | Many other industry participants - see the doc | <https://www.colegiodenutricionistas.cl/single-post/2019/08/28/IV-Curso-de-Nutricionistas-al-d%C3%ADa-de-Cl%C3%ADnica-Las-Condes> | 29-Nov-19 |
| A85 | Nestle | Profesionnal assoc | Information management | Amplification |  | X Congreso Chileno de Nutrición Clínica, Obesidad y Metabolismo, III Congreso de Nutrición Clínica Pediatrica  Viernes 26 de Abril 2019: 13:00 – 14:00 Simposio Lunch: "Preparo Inmunológico en el paciente quirúrgico" Expositor: Antonio Carlos Campos, Cirujano Digestivo, Coordinador del Programa de Post-Grado en Clínica Quirúrgica, Universidad Federal de Paraná, Curitiba, Brasil. Gentileza: Nestlé Health Science (cupos limitados) | Also sessions by pharma industry | <http://www.achinumet.cl/Congreso-2019/programa.html> | 04-Dec-19 |
| A86 | Nestle | Profesionnal assoc | Information management | Amplification |  | X Congreso Chileno de Nutrición Clínica, Obesidad y Metabolismo, III Congreso de Nutrición Clínica Pediatrica  Auspiciadores Diamante: Nestle Health Science | Among other sponsors - mostly pharma industry | <http://www.achinumet.cl/Congreso-2019/auspiciadores.html> | 04-Dec-19 |
| A87 | Nestle | University | Information management | Amplification |  | Las becas “INTA-Nestlé” tienen como finalidad contribuir al desarrollo académico e institucional del INTA, a través del apoyo económico de manutención a investigadores jóvenes que demuestren interés y potencial para ingresar a la carrera académica, promoviendo el compromiso con la investigación del instituto en áreas prioritarias.  Temas: Los temas prioritarios de investigación están relacionados con el desarrollo de las Ciencias en el ámbito de la nutrición y Tecnología de alimentos (Plan de Desarrollo Institucional INTA 2015-2015). Se valorarán los proyectos que contemplen actividades conjuntas entre más de un grupo académico del Instituto y/o con grupos de otras unidades académicas.  Dirigido a: Profesionales de nivel universitario con al menos grado de Magíster, candidato a Doctor y/o que posean la especialidad médica equivalente. |  | <https://inta.cl/convocatoria-becas-inta-nestle-2019/> | 05-Dec-19 |
| A88 | Nestle | University | Coalition management | Health organisations |  | INTA participa en instancia multisectorial de la ONU por el Hambre Cero Jueves 16 Mayo - 2019 En Septiembre del 2015 Chile, adoptó la Agenda 2030 para el Desarrollo Sostenible, impulsada por Pacto Global, iniciativa Organización de Naciones Unidas (ONU) que examina los temas críticos de Sostenibilidad en el mundo. (...) Este encuentro constó de una mesa de discusión para trabajar colaborativamente a ser un motor que revierta la dramática situación de la malnutrición en Chile. En la instancia se presentaron temáticas de acción de JUNAEB, EVS y se mostró el plan de trabajo para fomentar estilos de vida saludables. Estuvieron presente representantes de FAO, Corpora 3 Montes, Nestlé, MINAGRI, Sodexo, Universidad de Valpo, Universidad de Santiago, Alcaldía de María Pinto, entre otros. |  | <https://inta.cl/inta-participa-en-instancia-multisectorial-de-la-onu-por-el-hambre-cero/> | 05-Dec-19 |
| A89 | Nestle | University | Information management | Production |  | El Fondo de Desarrollo Local Premio Henri Nestlé 2019, beneficiará a organizaciones comunales de Antofagasta, Quilicura, Maipú, Macul, Graneros, Teno, San Fernando, Los Ángeles, Osorno y Llanquihue. El concurso tiene un especial enfoque este año en el medioambiente, haciendo un llamado a presentar soluciones relacionadas con el agua, la gestión de residuos y el desarrollo rural, sin dejar de lado proyectos de nutrición que tradicionalmente participan de este fondo.  Aquellos que estén buscando impactar positivamente a la sociedad y el medioambiente, y requieran un aporte de financiamiento para sus proyectos de sustentabilidad o nutrición, tienen una nueva oportunidad para impulsar sus innovaciones con el lanzamiento de la nueva edición del Fondo de Desarrollo Local Premio Henri Nestlé, presentado por la compañía suiza en Chile.(...) La empresa de alimentos ha abierto las postulaciones para este premio que se entrega hace más de 14 años en el país, y ya lleva cuatro versiones beneficiando a organizaciones comunales provenientes de los municipios donde Nestlé Chile opera a través de sus fábricas como Maipú, Macul, Graneros, San Fernando, Osorno, Los Ángeles, Llanquihue y Teno. |  | <https://www.nestle.cl/media/pressreleases/allpressreleases/%C2%A1inscribe-tu-proyecto-para-el-fondo-de-desarrollo-local-premio-henri-nestl%C3%A9> | 05-Dec-19 |
| A90 | Nestle | Government | Direct involvement and influence in policy Discursive | Lobby Frame the debate | Part of the solution | Datos de Audiencia Organismo público que informa: Subsecretaría De Salud Pública  Duración: 25 minuto(s)  Lugar: Santiago  Fecha Audiencia: 30 de julio de 2019 11:15 Observaciones: Temas a tratar: A la luz del plan de obesidad que lanzará el Gobierno, queremos presentar el programa educacional y nutricional "Niños Saludables" que ya impacta a 80 mil niños en Chile. Temas tratados: Vienen a mostrar su programa "Niños Saludables" de la empresa, dejan material que se usa en su plan educativo.  Lobbistas o Gestores de Interés  Nombre: Francisco Frei  Calidad: Lobista Nacionalidad: Chile Trabaja para: Nestle Chile  Persona o Entidad a la que Representa: Nestle Chile   Nombre: Ursula Preisler  Calidad: Lobista Nacionalidad: Chile Persona o Entidad a la que Representa: Nestle Chile |  | https://www.infolobby.cl/Ficha/Audiencia/ao0013663621 | 26-Dec-19 |
| A91 | Nestle | Twitter | Coalition management Information management Discursive | Community Amplification Frame the debate | Part of the solution (sustainibility) | Pacto Global Chile Compte certifié @PactoGlobal Suivre Suivre @PactoGloba Nuestra empresa adherida, @NestleCL, junto al Museo @artequin, inauguraron su Eco Zona, un espacio que busca generar conciencia en los niños sobre el cuidado del #MedioAmbiente a través del arte ✅ #TiempoDeActuar 🌎♻️ ➡️ Más información: http://ow.ly/LKxC50wOw1w | See pictures - branding Also retweeted by Ministry: https://twitter.com/medioambienterm/status/1184875665553838081 Promoted by the UN Global Compact: https://pactoglobal.cl/2019/eco-zona-de-nestle-uniendo-arte-y-medioambiente/ | <https://twitter.com/PactoGlobal/status/1185258053395984384> | 27-Dec-19 |
| A92 | Nestle | Twitter | Coalition management Discursive | Community Frame the debate | Part of the solution (sustainibility) | Nestlé Chile S.A. Compte certifié @NestleCL Suivre Suivre @NestleCL ¡EXTENDIMOS EL PLAZO! 👏 Última oportunidad para postular al Fondo de Desarrollo Local Premio Henri Nestlé 2019 y aportar positivamente a tu comunidad ✅💯. Tienes plazo hasta el 👉 21 de octubre, ¡no te quedes fuera! #Emprendedores #Sustentabilidad Más: https://bit.ly/2PfpxrY  09:00 - 18 oct. 2019 | Other tweets about the same contest | <https://twitter.com/NestleCL/status/1185224117190119424> | 27-Dec-19 |
| A93 | Nestle | Twitter | Coalition management Discursive | Community Frame the debate | Part of the solution Personal responsibility | @NestleCL Suivre Suivre @NestleCL Los educadores son un pilar fundamental en la alimentación saludable de los niños 👩‍🏫. En su día, agradecemos a todos los que han aportado con su entusiasmo a #Nestlé por #NiñosSaludables e invitamos a todos los profesores a contribuir a un futuro más saludable! 🍽. #HealthierKids 07:30 - 16 oct. 2019 |  | <https://twitter.com/NestleCL/status/1184476613209182208> | 27-Dec-19 |
| A94 | Nestle | Twitter | Discursive | Frame the debate | Part of the solution Personal responsibility | Diario Sustentable @diariosustentab Suivre Suivre @diariosustentab TVN (@TVN) y Nestlé (@NestleCL) se comprometen a llevar la bandera de una alimentación saludable en Chile http://blgs.co/61614u  09:00 - 14 oct. 2019 | See pictures - branding | <https://twitter.com/diariosustentab/status/1183774567795441665> | 27-Dec-19 |
| A95 | Nestle | Twitter | Information management | Production |  | Nestlé Chile S.A. ‏Compte certifié  @NestleCL Suivre Suivre @NestleCL ¡Conoce a las jóvenesque ganaron el Fondo de Desarrollo Científico SOCHINUT - Henri Nestlé! 🏅👩‍🔬 Cada una de ellas, a través de sus proyectos, buscan mejorar la nutrición de Chile 🍽. Más en: http://bit.ly/2p0zvRe . 15:00 - 9 oct. 2019 |  | <https://twitter.com/NestleCL/status/1182053214767722496> | 27-Dec-19 |
| A96 | Nestle | Twitter | Information management Discursive | Amplification Frame the debate | Part of the solution | @NestleCL Suivre Suivre @NestleCL Una de las formas de mejorar la calidad de vida y contribuir a un futuro más saludable es la investigación 👩‍🔬 que realizamos para comprender y proporcionar la nutrición adecuada en cada etapa de la infancia a través de nuestros productos 🍎 #NiñosSaludables #Nestlé #HealthierKids 08:00 - 25 sept. 2019 |  | <https://twitter.com/NestleCL/status/1176874139203883008> | 27-Dec-19 |
| A97 | Nestle | Twitter | Discursive | Frame the debate | Part of the solution | Nestlé Chile S.A. Compte certifié @NestleCL Suivre Suivre @NestleCl Durante el 2018 lanzamos más de 1.300 productos nuevos 👏 que abordan las distintas necesidades nutricionales con el objetivo de entregar alimentos más nutritivos a quienes los necesitan 🍎🍐🌾🥛🍽. 08:00 - 28 août 2019 |  | <https://twitter.com/NestleCL/status/1166727183324332032> | 27-Dec-19 |
| A98 | Nestle | Twitter | Coalition management Discursive | Community Frame the debate | Part of the solution | Nestlé Chile S.A.‏ Compte certifié @NestleCL Suivre Suivre @NestleCL #CambiarEsNatural cuando nos alimentamos directamente desde el huerto 🌾. A través del proyecto comunitario Huertos Familiares 🌱, @Maggi_Chile promueve el consumo de verduras y fomenta estilos de vida más saludables 🍊🍐🍎. 10:52 - 10 mai 2019 | See pictures - branding | <https://twitter.com/NestleCL/status/1126907905385598976> | 27-Dec-19 |
| A99 | Nestle | Twitter | Discursive | Frame the debate | Part of the solution Personal responsibility | Nestlé Chile S.A. ‏Compte certifié  @NestleCL Suivre Suivre @NestleCL Centramos nuestro trabajo en ofrecer alternativas más deliciosas y saludables, inspirar a las personas a tener vidas más saludables y a construir, compartir y aplicar el conocimiento nutricional. Conoce nuestros compromisos en: http://goo.gl/ZgNEn2  08:00 - 5 avr. 2019 | Other similar tweets: https://twitter.com/NestleCL/status/1105121344520421376 https://twitter.com/NestleCL/status/1102244767595941890 https://twitter.com/NestleCL/status/1098346162271240192 https://twitter.com/NestleCL/status/1085998952745484289 https://twitter.com/NestleCL/status/1085197459037540352 https://twitter.com/NestleCL/status/1083098608835219467 https://twitter.com/NestleCL/status/1081211175759740930 | <https://twitter.com/NestleCL/status/1114180916509851648> | 27-Dec-19 |
| A100 | Nestle | Twitter | Discursive | Frame the debate | Part of the solution Parental responsibility | @NestleCL Suivre Suivre @NestleCL Conoce 5 formas de alimentar a tu hijo de forma que crezca fuerte y saludable. Ingresa a: http://goo.gl/8tkEpc #NiñosSaludables #Nestlé #HealthierKids 🍽 14:30 - 12 mars 2019 |  | <https://twitter.com/NestleCL/status/1105581765157154816> | 27-Dec-19 |
| A101 | Nestle | Twitter | Discursive | Frame the debate | Part of the solution Personal responsibility, healthy lifestyles etc | Nestlé Chile S.A. ‏Compte certifié  @NestleCL Suivre Suivre @NestleCL Más de 40 millones de niños menores de 5 años tienen sobrepeso o son obesos. Ayudando a las nuevas generaciones a llevar estilos de vida más saludables, mejoraremos la calidad de vida y contribuiremos a un futuro más saludable. Más información en: http://goo.gl/dXhMvX 🍽 14:00 - 8 mars 2019 |  | <https://twitter.com/NestleCL/status/1104139766902202368> | 27-Dec-19 |
| A102 | Nestle | Twitter | Discursive | Frame the debate | Part of the solution History in the country | @NestleCL Suivre Suivre @NestleCL Desde 1934 estamos presentes en Chile, con el objetivo de contribuir a un futuro más saludable. Conoce uno de nuestros esfuerzos por cumplir nuestro objetivo en: http://goo.gl/HBUHWy 🥛 07:30 - 6 mars 2019 |  | <https://twitter.com/NestleCL/status/1103316845917077504> | 27-Dec-19 |
| A103 | Nestle | Twitter | Discursive | Frame the debate | Part of the solution | @NestleCL Suivre Suivre @NestleCL Guiados por nuestros valores, trabajamos para mejorar la calidad de vida y contribuir a un futuro más saludable. Conoce más sobre nuestro propósito en: http://goo.gl/T9uMFF  14:16 - 20 févr. 2019 |  | <https://twitter.com/NestleCL/status/1098345647437238272> | 27-Dec-19 |
| A104 | Nestle | Twitter | Information management | Amplification |  | Nestlé Chile S.A. Compte certifié @NestleCL Suivre Suivre @NestleCL Una adecuada nutrición durante los primeros 1000 días de vida de un bebé es esencial. Aprende cómo desarrollar hábitos alimentarios saludables para toda la familia en: http://goo.gl/dif6kh  08:00 - 13 févr. 2019 |  | <https://twitter.com/NestleCL/status/1095714269759528962> | 27-Dec-19 |
| A105 | Nestle | Twitter | Information management | Amplification |  | @NestleCL Suivre Suivre @NestleCL La Guía de Actividades en Alimentación y Nutrición para el Docente es la base del programa Nestlé por #NiñosSaludables, la cual busca promover hábitos de alimentación saludable a través de la educación nutricional. Conoce más en: http://goo.gl/nU37t4 #HealthierKids #Nestlé 10:00 - 5 févr. 2019 |  | <https://twitter.com/NestleCL/status/1092845332780716033> | 27-Dec-19 |
| A106 | Nestle | Twitter | Discursive | Frame the debate | Part of the solution Parental responsibility | Nestlé Chile S.A. Compte certifié @NestleCL Suivre Suivre @NestleCL Disfruta del verano de manera saludable junto a tus hijos. Conoce algunos tips saludables para estas vacaciones en: http://goo.gl/uvE8oz  06:30 - 2 févr. 2019 |  | <https://twitter.com/NestleCL/status/1091705325630251008> | 27-Dec-19 |
| A107 | Nestle | Twitter | Discursive | Frame the debate | Self-regulation Part of the solution | Nestlé Chile S.A. Compte certifié @NestleCL Suivre Suivre @NestleCL Nuestros productos incluyen en sus envases la Guía Diaria de Alimentación, la cual detalla el aporte nutricional de una porción. Conoce más de nuestros compromisos en nutrición en: http://goo.gl/xJ6Mzm  07:30 - 8 janv. 2019 |  | <https://twitter.com/NestleCL/status/1082660730086871041> | 27-Dec-19 |
| A108 | Nestle | Twitter | Coalition management Information management Discursive | Professionnal organisations Production Frame the debate | Part of the solution | Jóvenes científicas que buscan mejorar la nutrición de Chile fueron premiadas con el Fondo de Desarrollo Científico SOCHINUT – Henri Nestlé Entre los proyectos ganadores se encuentra un estudio sobre el impacto de las dietas altas en grasas saturadas en el cerebro, otro sobre el consumo de la sal yodada y uno en torno al estado nutricional de lactantes y preescolares chilenos. Oct 07, 2019 Tres jóvenes científicas fueron recientemente premiadas con el Fondo de Desarrollo Científico SOCHINUT – Henri Nestlé, un apoyo financiero que les permitirá impulsar sus proyectos que buscan mejorar la salud y calidad de vida de los chilenos a través de la nutrición y la alimentación. (...) En los casi 15 años de trayectoria que tiene el reconocimiento SOCHINUT - Henri Nestlé se han destacado a cerca de 30 investigadores, cuyos proyectos han contribuido a tener una mayor y mejor información sobre las propiedades nutricionales de los alimentos y sus potencialidades en la prevención de algunas enfermedades. En esta oportunidad, los proyectos fueron evaluados por un comité de jueces compuesto por el comité de expertos, el directorio de SOCHINUT y un comité de expertos de Nestlé. La ceremonia de premiación contó con la presencia de representantes del mundo de la nutrición y la salud. En la ocasión, Úrsula Preisler, Gerente de Nutrición, Salud y Bienestar de Nestlé Chile, destacó la importancia de la ciencia para mejorar la nutrición de los chilenos. “Con el Fondo de Desarrollo Científico SOCHINUT – Henri Nestlé buscamos apoyar el espíritu innovador de las investigaciones que buscan mejorar la salud de los chilenos a través de la nutrición y la alimentación. Es de este modo como damos continuidad a uno de los ejes fundacionales de Nestlé, enfocado en la innovación basada en principios científicos para así mejorar la calidad de vida y contribuir a un futuro más saludable para las personas y sus familias”. |  | <https://www.nestle.cl/media/pressreleases/allpressreleases/j%C3%B3venes-cient%C3%ADficas-que-buscan-mejorar-la-nutrici%C3%B3n-de-chile-fueron-premiadas> | 27-Dec-19 |
| A109 | Nestle | Website industry | Information management | Amplification |  | Nutrición en bebes ​​5 formas de impulsarlos a ser niños sanos y fuertes  ​​Todos queremos que nuestros niños crezcan lo más fuertes y saludables posible, y para darles el mejor comienzo en la vida significa darles alimentos indicados.​ Pero se sabe que es más fácil decirlo que hacerlo, particularmente con niños pequeños. Hay mucho que puede hacer para incentivar a los niños a comer bocadillos y comidas balanceadas y nutritivas.  Aquí hay cinco consejos de cómo mantener a los pequeños en el camino nutricional correcto…  1.- Aumentar su ingesta de hierro 2.- Darles un arcoíris de vegetales 3.- Incluir muchos granos enteros 4.- Tomar leche 5.- Hágase amigo de ese gusto dulce | See other webpages with advice in nutrition: https://www.nestle.cl/historias/snacks-saludables https://www.nestle.cl/historias/investigacion-epigenetica-antepasados-alimentacion-afecta-salud https://www.nestle.cl/historias/la-dieta-desconecta-cmo-se-ajustan-los-comportamientos-alimentarios-y-de-actividad-de-los-nios  FITS study funded by Nestlé from the US is mentioned (see our paper on Nestlé and baby food industry in the USA) https://www.nestle.cl/historias/sabroso-y-saludable-5-consejos-para-que-la-buena-nutricin-sea-un-asunto-familiar | <https://www.nestle.cl/historias/nutricion-en-bebes> | 31-Dec-19 |
| A110 | Nestle | Website industry | Discursive | Frame the debate | Part of the solution Self-regulation | Como parte de nuestro compromiso con mejorar contantemente el perfil nutricional de nuestros productos, Nestlé ha estado reduciendo el azúcar en muchos productos, especialmente los productos infantiles. Entre 2000 y 2013 hemos reducido la cantidad de azúcar libre en nuestros productos en un 32%.  El azúcar libre es definido por la Organización Mundial de la Salud como "todos los monosacáridos y disacáridos añadidos a los alimentos por el fabricante, cocinero o consumidor, además de los azúcares normalmente presentes en miel, jarabes y jugos de frutas". Por lo tanto, el azúcar que se le añade al té, por ejemplo, es llamado azúcar libre. Por ejemplo, en 2016 redujimos en Chile un 20,2% el nivel de sodio de nuestros helados y en un 5,7% el de nuestros productos culinario. |  | <https://www.nestle.cl/faq/que-estan-haciendo-para-reducir-la-cantidad-de-azucar-en-sus-productos> | 31-Dec-19 |
| A111 | Nestle | Website industry | Information management Discursive | Amplification Frame the debate | Physical activity Personal responsibility | ¡REALIZA MÁS ACTIVIDAD FÍSICA Y SÉ MÁS FELIZ! | Multiple webpages and brochures on the themes of nutrition and physical activity: https://www.nestle-contigo.cl/sites/default/files/pdf/nutrigroup/educativo/embarazo.pdf https://www.nestle-contigo.cl/sites/default/files/pdf/nutrigroup/educativo/actividad_fisica.pdf https://www.nestle-contigo.cl/index.php/bienestar-y-nutricion/articulos/conoce-los-beneficios-del-aceite-de-coco https://www.nestle-contigo.cl/sites/default/files/pdf/nutrigroup/educativo/cuida_tu_salud.pdf https://www.nestle-contigo.cl/bienestar-y-nutricion/articulos/prepara-estas-ricas-recetas-con-palta-y-cuida-tu-salud | <https://www.nestle-contigo.cl/bienestar-y-nutricion/articulos/realiza-mas-actividad-fisica-y-se-mas-feliz> | 31-Dec-19 |
| A112 | Nestle | Website industry | Information management Discursive | Amplification Frame the debate | Part of the solution Education | Nestlé Niños Saludables es un programa global de nuestra compañía que se desarrolla desde 2009 y ya está presente en más de 80 países. Tiene como objetivo promover estilos de vida saludables en niños, preparando y empoderando a quienes los cuidan. Llega a más de 8 millones de niños en el mundo.  En Chile, el programa busca promover hábitos de alimentación sana a través de la educación nutricional temprana en la sala de clases. Para lograrlo, se capacita a profesores de pre kínder a 4o básico, para que ellos realicen actividades educativas en las aulas con sus alumnos con el soporte de nutricionistas del programa.  El programa partió en 2010 en la comuna de Peñalolén en 9 colegios municipales. Debido al éxito de este piloto, se fue extendiendo a más comunas del país, -actualmente 53-, lo que nos permite beneficiar a más de 63.000 niños que reciben educación nutricional impartida por sus propios profesores. A julio de 2018, Nestlé Niños Saludables ha capacitado a más de 1.500 profesores de 237 colegios del país, distribuidos de Arica a Punta Arenas, e incluyendo Isla de Pascua.  Nestlé desarrolló los contenidos del programa y todo el material educativo de uso en la sala desde pre kínder a 4 año básico con un equipo de nutricionistas. Se estructuró sobre la base de 6 ejes temáticos: "Colaciones", "Pescado", "Legumbres", "Lácteos", "Frutas y Verduras" y "Agua", basados en las Guías Alimentarias para la Población Chilena del Ministerio de Salud.  Todos los contenidos se reúnen en una Guía Didáctica para el Docente y fueron revisados y validados por el Instituto de Nutrición y Tecnología de los Alimentos de la Universidad de Chile (INTA), la Fundación 5 al Día y la Sociedad Chilena de Nutrición (SOCHINUT). Asimismo, desde 2013, anualmente esta publicación ha recibido el patrocinio del Ministerio de Educación.  Tras capacitar a los profesores, Nestlé entrega todo el material necesario para realizar las actividades educativas en la sala de clases. Un nutricionista del equipo de 12 profesionales de terreno acompaña y apoya al profesor en la realización de la actividad educativa, dentro de la sala de clase, en un mínimo de 6 instancias anuales, una por cada eje. (...)   El programa Nestlé Niños Saludables cuenta con el patrocinio de tres importantes instituciones, que conjuntamente revisaron y validaron los contenidos de esta Guía: El INTA, Fundación 5 al Día y la Sociedad Chilena de Nutrición (SOCHINUT).  Desde 2013, el material educativo cuenta con el patrocinio del Ministerio de Educación, el que se renueva anualmente.  El programa no está relacionado con ninguna marca. Tanto en Chile, como en el resto del mundo, se trata de un programa que no promociona -bajo ninguna forma ni contexto- marcas o productos de nuestra compañía.   Como compañía, creemos que podemos y debemos ser parte de la solución al problema de obesidad infantil que enfrenta Chile. Y esto no sólo lo hacemos ofreciendo las opciones más ricas y nutritivas en cada una de las categorías en las que estamos presentes, gracias a la mejora nutricional continua de nuestro portafolio, sino también aportando con todo nuestro expertise nutricional en el ámbito educativo para la formación de hábitos. Creemos que la obesidad es un problema multifactorial que requiere respuestas multifactoriales e intentaremos contribuir en todos los ámbitos que podamos hacer un aporte.​​​ |  | <https://www.nestle.cl/faq/programa-nestle-ninos-saludables> | 31-Dec-19 |
| A113 | PepsiCo | Twitter | Discursive | Frame the debate | Part of the solution Personal responsibility | @PepsiCoChile Suivre Suivre @PepsiCoChile Estamos comprometidos con mejorar nuestro portafolio de productos y ofrecer más opciones a los consumidores, reduciendo ingredientes como azúcares añadidos, sodio y grasas saturadas. 13:00 - 10 déc. 2019 | See also: https://twitter.com/PepsiCoChile/status/1161336671176155137 | <https://twitter.com/PepsiCoChile/status/1204506113963679744> | 27-Dec-19 |
| A114 | PepsiCo | Twitter | Discursive | Frame the debate | Part of the solution | PepsiCo Chile ‏@PepsiCoChile Nos preocupamos impulsar la conservación del agua para asegurar la continuidad de nuestro negocio, a la vez que contribuimos positivamente a las comunidades en donde operamos. 0:03 10:00 - 1 déc. 2019 | Much messages about the company and its efforts in sustainibility | <https://twitter.com/PepsiCoChile/status/1201199370458320896> | 27-Dec-19 |
| A115 | PepsiCo | Twitter | Information management Discursive | Amplification Frame the debate | Part of the solution | PepsiCo Chile @PepsiCoChile Suivre Suivre @PepsiCoChile Fundación #PepsiCo, junto a @TriCiclosB, trabaja en educación medioambiental en ocho colegios públicos de Cerrillos. El Programa “Mis residuos, Mi responsabilidad” ha reciclado más de 3 mil 500 kg junto a los colegios que participan. 08:00 - 13 oct. 2019 |  | <https://twitter.com/PepsiCoChile/status/1183397004237520896> | 27-Dec-19 |
| A116 | PepsiCo | Twitter | Coalition management Discursive | Ministry of Sport Community Frame the debate | Part of the solution (recycling) | PepsiCo Chile @PepsiCoChile Suivre Suivre @PepsiCoChile A través de una alianza con el @MindepChile, Gatorade donó 300 kits de entrenamiento a colegios del país. Cada uno, compuesto por conos, aros y escalerillas fabricados a partir de vasos reciclados de los mismos puntos de hidratación de la bebida. 07:49 - 10 oct. 2019 (...) Santiago Escobar, director de Bebidas de PepsiCo Chile, resaltó la importancia de la iniciativa ya que nuestra compañía “buscar ser parte de construir un mundo en el que los plásticos nunca se conviertan en desechos, y esto es un ejemplo puntual de cómo conseguirlo”. (...) La ceremonia de entrega de los utensilios contó con la presencia de la ministra del Deporte @kantor_pauline y destacados deportistas nacionales. | See pictures - branding | <https://twitter.com/PepsiCoChile/status/1182307184929132545> | 27-Dec-19 |
| A117 | PepsiCo | Website industry | Coalition management | Community |  | PepsiCo Chile trabaja en alianza y en sinergia con diversas instituciones en variadas temáticas de interés social:  ActivaRSE Mi Escuela, Mi Mundo Educación para el Desarrollo Avanza Puntos Limpios Vive Saludable |  | <http://www.pepsico.cl/purpose/pepsico-foundation> | 31-Dec-19 |
| A118 | PepsiCo | Website industry | Coalition management Information management Discursive | Community Amplification Frame the debate | Physical activity Personal responsibility | Brinda a la comunidad educativa herramientas de acción y concientización para la promoción de una vida saludable, teniendo como eje principal el desarrollo integral de la infancia.  Son destinatarios del Programa alumnos de entre 6 y 9 años que estén cursando 1º, 2º y 3º grado; sus directivos, docentes y familias.  A través de Jornadas de capacitación a docentes y padres, talleres para niños y la entrega de material deportivo, se implementan propuestas didácticas que permitan abordar temáticas relacionadas con los siguientes ejes fundamentales: Educación en Nutrición, Importancia de realizar Actividad física, Establecimiento de Hábitos saludables | See pictures: branding | <http://www.pepsicocomunidad.cl/activarse.php> | 31-Dec-19 |
| A119 | PepsiCo | Website industry | Coalition management Discursive | Community Frame the debate | Part of the solution Personal responsibility | Mi escuela, mi mundo, es un programa de Fundación PepsiCo diseñado para promover el cuidado del medio ambiente a través de la educación y de la participación ciudadana por parte de los jóvenes en las comunidades donde PepsiCo está presente. (….) Beneficiarios directos: Estudiantes de escuelas secundarias públicas de Vicente López, Quilmes y Mar del Plata, equipo docente y equipo directivo de éstas escuelas ubicadas en comunidades en las cuales PepsiCo está presente. |  | <http://www.pepsicocomunidad.cl/mi-escuela-mi-mundo.php> | 31-Dec-19 |
| A120 | PepsiCo | Website industry | Coalition management Information management Discursive | Community Amplification Frame the debate | Personal responsibility | Los Talleres Vive Saludable proponen un espacio de formación y participación destinado a niños y niñas de 1º a 4º básico, de escuelas de toda la región Metropolitana.  A través de una obra de teatro, lúdica y divertida, los Talleres Vive Saludable buscan transmitir conceptos fundamentales para el desarrollo de los niños:  Concientizar sobre la adquisición de hábitos alimentarios más saludables, incorporando una elección adecuada de los alimentos. Promover la incorporación de la actividad física y el movimiento para la obtención de una mejor calidad de vida. |  | <http://www.pepsicocomunidad.cl/talleres-vive-saludable.php> | 31-Dec-19 |
| A121 | PepsiCo | Website industry | Coalition management Discursive | Community Frame the debate | University Part of the solution | Juguemos con Nuestros Hijos  El objetivo del programa se centra en entregar las herramientas necesarias para que padres y madres de sectores vulnerables puedan estimular tempranamente a sus hijos, a través del desarrollo de elementos psicomotores fundamentales como la comunicación, la lectura dialogada, y los juegos de motivación.  Se desarrolla en articulación con la Universidad Católica de Chile y está dirigido a niños de 0 a 4 años de edad. |  | <http://www.pepsicocomunidad.cl/juguemos-con-nuestros-hijos.php> | 31-Dec-19 |
| A122 | PepsiCo | Website industry | Information management Discursive | Amplification Frame the debate | Part of the solution Personal responsibility | PepsiCo forma parte de NutriRSE, un proyecto asociativo entre empresas, organismos públicos y técnico-académicos.  El objetivo es promover los estilos de vida integral y saludable, a través de la promoción de una alimentación equilibrada, actividad física y el cuidado de la salud mental. |  | <http://www.pepsicocomunidad.cl/nutrirse.php> | 31-Dec-19 |
| A123 | PepsiCo | Website industry | Coalition management |  |  | Partner y Aliados Partner www.casadelapaz.cl Espacios de Responsabilidad Social Corporativa www.accionrse.cl Otras instituciones www.fao.org/home/es  www.iadb.org  www.corfo.cl  www.eligevivirsano.cl  www.gob.cl  www.uc.cl  www.techo.org  web.redalimentos.cl  www.gestionsocial.cl |  | <http://www.pepsicocomunidad.cl/partner-aliados.php> | 31-Dec-19 |
| A124 | PF Alimentos | Profesionnal assoc | Information management | Amplification |  | 8° Simposio de Enfermedad Celíaca September 16, 2019 Organizan: COACEL, INTA Participan: … PF Alimentos… | Many other industry participants - see the doc | <https://www.colegiodenutricionistas.cl/single-post/2019/09/16/8%C2%B0-Simposio-de-Enfermedad-Cel%C3%Adaca> | 29-Nov-19 |
| A125 | Soprole | Website industry | Coalition management Discursive | Community Frame the debate | Physical activity Personal responsibility Part of the solution | Deporte Escolar Soprole mantiene un compromiso de largo plazo con la familia chilena, incentivando la actividad física y la práctica de deportes. Por eso, como padres, apoyamos la Vida Saludable a través del programa de Deporte Escolar más masivo del país, que reúne cada año, a más de 2 millones de personas, niños y sus familias, de Arica a Punta Arenas.  Alimentación Saludable Nuestros hijos son la razón de todo lo que hacemos. Ellos nos dan la fuerza que hace que todos los chilenos nos levantemos día a día. Todos los que trabajamos en Soprole tenemos familia y gran parte de nosotros somos padres, con las mismas preocupaciones y motivaciones sobre nuestros hijos; por eso, cada día ponemos el máximo esfuerzo en desarrollar productos Sanos y Ricos, para alimentar a los niños de nuestro país, nuestros propios hijos.  Teletón  Este año, Soprole cumplirá 40 años de apoyo ininterrumpido a la Fundación Teletón, la obra privada más importante de Chile en favor de los niños con capacidades diferentes. Desde su inicio, en 1978, Soprole ha participado con sus marcas y con sus trabajadores, en un compromiso real por la rehabilitación de los niños; una conexión y empatía tan marcada con el espíritu de Soprole, que ha sido elegida por el público como la empresa más identificada con la Teletón. |  | <https://www.soprole.cl/es/nosotros/social-responsibility.html> | 31-Dec-19 |
| A126 | Soprole Nestle | Other | Coalition management | Internal |  | El Consorcio Lechero es una Sociedad Anónima conformada por las principales industrias lecheras que trabajan en el país, así como por los productores lecheros, junto a las más importantes entidades tecnológicas y de servicio del sector lácteo. COLUN SOPROLE WATT'S NESTLE PROLESUR APROVAL SAGO AG COOPRINSEM VETERQUIMICA ANASAC BEST-FED ABS CHILE SHOOF UNIVERSIDAD AUSTRAL DE CHILE INIA |  | <https://consorciolechero.cl/nuestros-socios/> | 27-Dec-19 |
| A127 | Soprole Nestle | Profesionnal assoc | Information management | Amplification |  | 3° versión Curso lácteos, nutrición y salud ¿Qué nos dice la evidencia científica? October 14, 2019, Faculdad de Medicina, Universidad de Chile Organiza: Consorcio Lechero, Departamento de Nutricion, Faculdad de Medicina, Universidad de Chile Patrocinado: Colegio de Nutricionistas Universitarios de Chile A.G, Sociedad Chilena de Nutricion |  | <https://www.colegiodenutricionistas.cl/single-post/2019/10/14/3%C2%B0-versi%C3%B3n-Curso-l%C3%A1cteos-nutrici%C3%B3n-y-salud-%C2%BFQu%C3%A9-nos-dice-la-evidencia-cient%C3%Adfica> | 29-Nov-19 |
| A128 | Soprole Nestle | Profesionnal assoc | Information management | Amplification |  | JUN.03 Curso: Lácteos, Nutrición y Salud de Consorcio Lechero Universidad de Concepcion, Consorcio Lechero |  | [https://www.eventbrite.es/e/registro-curso-lacteos-nutricion-y-salud-59850560638#](https://www.eventbrite.es/e/registro-curso-lacteos-nutricion-y-salud-59850560638) | 29-Nov-19 |
| A129 | Soprole Nestle | University | Information management | Production |  | INTA y el Consorcio Lechero firmaron convenio de colaboración Viernes 11 Octubre - 2019 \| Categoría Noticias La alianza busca potenciar las actividades y capacidades técnicas de cada institución, para promover los beneficios de la leche entre consumidores, profesionales de la salud y autoridades, a través de proyectos de investigación y extensión entre otros. Con el objetivo claro de potenciar los lácteos como un alimento nutritivo y saludable, el Instituto de Nutrición y Tecnología de los Alimentos de la Universidad de Chile (INTA) y el Consorcio Lechero, que involucra a una gran asociación de productores pequeños, medianos y grandes, firmaron un convenio de colaboración, que contempla un trabajo de investigación y extensión en conjunto, así como la promoción de los lácteos en la dieta de los chilenos. Sobre la relevancia de este acuerdo el director del INTA, profesor Francisco Pérez, dijo: “Consideramos que es importante, en primer lugar, por el valor nutricional que tiene la leche y sus derivados en la alimentación de los chilenos, y porque es un convenio amplio de colaboración que permitirá investigar y difundir estos hallazgos a gran escala, donde la experiencia y el renombre del INTA tiene mucho que decir al respecto”. “La leche es un alimento que ha estado presente siempre en los hogares chilenos. Es energía que nos mueve, y así la asociamos a una nutrición completa y como un elemento fundamental para el desarrollo de los niños y adolescentes, entre otras cualidades. Queremos que eso no se pierda, y revindicar las inmensas propiedades nutritivas de los lácteos, que junto con una industria sustentable, como la que promovemos desde el Consorcio, transforman a la leche en una herramienta clave para la salud pública”, afirmó Claudio Sarah, presidente del Consorcio Lechero de Chile. Esta alianza público-privada, refleja el interés de generar instancias que permitan dar a conocer los beneficios de la leche, entre consumidores, profesionales de la salud y autoridades, a través de la cooperación entre ambas entidades. “Ante el preocupante aumento de enfermedades como la obesidad, la diabetes o las cardiovasculares, los lácteos aparecen como un alimento de fácil acceso para la mayoría de las personas, de consumo rápido y fácil en sus diferentes presentaciones. Leche natural y saborizada, yogur, queso, son opciones que deben ser parte de una alimentación equilibrada y saludable”, afirmó Carmen Gloria Yáñez, directora de Asistencia Técnica del INTA. |  | <https://inta.cl/12585-2/> | 05-Dec-19 |
| A130 | Unilever | Website industry | Discursive | Frame the debate | Part of the solution Self-regulation | Vamos a seguir trabajando para mejorar el sabor y la calidad nutricional de todos nuestros productos. La mayoría de nuestros productos alcanzan o superan los valores de referencia según las recomendaciones nutricionales nacionales. Nuestro compromiso va más allá: para el año 2020, duplicaremos la proporción de nuestro portfolio que se ajusta a los más altos estándares nutricionales, según las directrices alimentarias reconocidas a nivel mundial. Esto contribuirá a que cientos de millones de personas puedan seguir una dieta más saludable. En el año 2018, el 48 % de nuestro portfolio se ajustó a los más altos estándares nutricionales |  | <https://www.unilever.cl/sustainable-living/> | 31-Dec-19 |
