# Supplementary material for: Food industry political practices in Chile: “the economy has always been the main concern”
Source: Global Health. 2020 Oct 27;16:107. doi: 10.1186/s12992-020-00638-4 (PMC7590241; doi:10.1186/s12992-020-00638-4)
Supplement: Supplementary file 4 — Additional file 4. Interview guide (Spanish). [file 12992_2020_638_MOESM4_ESM.docx]

Appendix 4: Interview guide (Spanish)

**Introducción (inicio de cada entrevista):**

Gracias por aceptar participar en este proyecto de investigación. Sé que su tiempo es extremadamente precioso, y gracias por usarlo para contribuir con este importante proyecto.

Como mencioné en mi carta de invitación, el objetivo de esta investigación es comprender las prácticas de la industria de alimentos que pueden influenciar la prevención y el control de las enfermedades crónicas no transmisibles relacionadas con la alimentación. El contexto es que el poder y la influencia de la industria de alimentos se han identificado como un factor importante que influye en las políticas de salud pública y la opinión pública en todo el mundo. Nuestro objetivo es identificar y monitorear esas prácticas, como parte de los esfuerzos para prevenir y controlar las enfermedades crónicas no transmisibles relacionadas con la alimentación. Usted ha sido seleccionado debido a su amplia experiencia de primera mano, observando y / o interactuando con la industria de alimentos, y creemos que puede proporcionar una visión crítica de las prácticas de la industria de alimentos. Hablaré con una serie de expertos, como usted, para obtener una visión detallada de esta área.

Espero que la entrevista lleve unos 45 minutos a 1 hora. Si usted está de acuerdo, escribiré la entrevista para cerciorarse de que registro sus puntos de vista con precisión, pero tenga el 100% de garantía de que toda la información proporcionada en la entrevista no será vinculada a su nombre y no será identificable como proporcionada por usted.

Firmar el formulario de consentimiento (entrevista + grabación digital)

*Perguntas-chave Prompts (se necessário)*

- ¿Para empezar, usted podría decirme de qué manera interactuó con la industria alimenticia, como parte de su profesión?

*Trabajo actual, trabajos anteriores, reuniones, alianzas, fondos*

- Y, específicamente, en qué roles profesionales y en qué situaciones interactuaste y observó la industria de alimentos?
- Como he mencionado, ciertas prácticas de la industria alimentaria están potencialmente proyectadas para influir en la política de salud pública. ¿Cuáles prácticas ha observado al respecto?

*exemplos*

- Ahora voy a presentar una lista de prácticas de la industria de alimentos que fueron previamente identificadas como potencialmente influyentes en la prevención y control de las Enfermedades Crónicas No Transmisibles relacionadas a la alimentación. Para cada una de las prácticas, usted puede indicar si usted la experimentó o observó?
- ¿Podrías decirme cuáles de estas prácticas crees que pueden representar un riesgo para las políticas y resultados de salud pública y por qué?
- ¿Podría indicar cuáles de estas prácticas creen más influyentes en las políticas y los resultados de la salud pública? Y cuáles son los menos influyentes
- Por último, nos gustaría entrevistar a otras personas que puedan proporcionar información sobre esta área. ¿Quién más usted sugiere que entrevistamos sobre eso?

*por ejemplo. ex políticos, ex-personas de la industria alimentaria, otras personas que tienen una vasta experiencia en esta área*

*Gracias de nuevo por su importante visión sobre esta área y por ofrecer su tiempo para ayudar en este proyecto de investigación. Explique cómo voy a comunicar los resultados con ellos y los próximos pasos.*

Interview guide (English)

**Introduction (beginning of each interview):**

Thank you for agreeing to participate in this research project. I know that your time is extremely precious, so thank you for using it to contribute to this important project.

As I mentioned in my invitation letter, the goal of this research is to understand the food industry practices that can influence the prevention and control of diet-related non-communicable diseases. The context is that the power and influence of the food industry has been identified as a major factor influencing public health policies and public opinion around the world. Our goal is to identify and monitor these practices, as part of our efforts to prevent and control non-communicable diseases related to food consumption. You have been selected because of your extensive first-hand experience, observing and / or interacting with the food industry, and we believe that you can provide critical insight into food industry practices. I will speak with a number of experts, like you, to get a detailed look at this area.

I expect the interview to take about 45 minutes to 1 hour. If you agree, I will take notes to make sure I record your views accurately, but be 100% guaranteed that all information provided in the interview will not be linked to your name and will not be identifiable as provided by you.

Sign the consent form (interview + digital recording)

*Key questions Prompts (where necessary)*

- To start with, could you tell me how you interacted with the food industry as part of your profession?

*Current job, previous jobs, meetings, alliances, funding*

- And, specifically, in what professional roles and in what situations did you interact and observe the food industry?
- As I mentioned, certain practices in the food industry are potentially developed to influence public health policy. What practices have you observed in this regard?

*Examples*

- I am now going to present a list of food industry practices that were previously identified as potentially influential in the prevention and control of food-related Noncommunicable Diseases. For each of the practices, can you indicate whether you experienced or observed it?
- Could you tell me which of these practices you think may pose a risk to public health policies and outcomes and why?
- Could you indicate which of these practices you believe to be most influential on public health policies and outcomes? And which are the least influential
- Finally, we would like to interview other people who can provide information on this area. Who else do you suggest we interview about that?

*For example. ex-politicians, former food industry employees, other people who have vast experience in this area*

*Thanks again for your important insight into this area and for volunteering your time to assist with this research project. Deatils of how I will communicate the results with them and the next steps.*
